# Identification of antimalarial drugs and bradykinin as ligands of the *Plasmodium* membrane protein PfSR10

**DOI:** 10.1016/j.isci.2025.113807

**Published:** 2025-10-21

**Authors:** Dan Jiang, Xin Wen, Ji-Fei Han, Qiu-Xia Cheng, Ru Zhang, Yuan Zheng, Kai Zheng, Shao-Hui Huang, Jia-Yuan Chen, Su-Wen Li, Zhi-Shuai Yang, Bing Han, Lu Tie, Fan Yang, Peng-Ju Zhang, Peng Xiao, Hui Lin, Xiao Yu, Jin-Peng Sun

**Affiliations:** 1 New Cornerstone Science Laboratory, Department of Biochemistry and Molecular Biology, School of Basic Medical Sciences, Cheeloo College of Medicine, Shandong University, Jinan, Shandong 250012, China; 2 Department of Physiology, School of Basic Medical Sciences, Cheeloo College of Medicine, Shandong University, Jinan, Shandong 250012, China; 3 Department of Physiology and Pathophysiology, School of Basic Medical Sciences, Peking University, Key Laboratory of Molecular Cardiovascular Science, Ministry of Education, Beijing 100191, China; 4 Department of Pathogenic Biology, School of Basic Medical Sciences, Cheeloo College of Medicine, Shandong University, Jinan, China; 5 Department of Biophysics, School of Basic Medical Sciences, Peking University Health Science Center, Beijing 100191, China; 6 NHC Key Laboratory of Otorhinolaryngology, Qilu Hospital of Shandong University, Jinan, Shandong 250012, China

**Keywords:** Natural sciences, Biological sciences, Biochemistry, Microbiology

## Abstract

Malaria caused by *Plasmodium falciparum* remains a public health issue, yet direct targets of antimalarial drugs remain elusive. Membrane proteins in *Plasmodium* are potential drug targets and may contribute to pathophysiological processes in malaria. Recent studies show that the serpentine receptor SR10 is essential for coordinating host rhythms during parasite development. In this study, we found that antimalarial drugs including chloroquine (CQ), dihydroartemisinin (DHA), piperaquine-tetraphosphatetetrahydrate (PIP-TT), and primaquine diphosphate (PQ) are PfSR10 agonists that induce coupling with human Gi/Gq proteins, confirmed through biochemical reconstitution and cryo-EM analysis. Using proteomic profiling, we also identified bradykinin as an endogenous agonist activating PfSR10. Ligand binding and conformational changes were characterized via mutagenesis and FlAsH-BRET assays. These results establish PfSR10 as a receptor for both antimalarials and host peptides, highlighting its dual role in drug action and host-parasite communication, with broad implications in understanding malaria pathogenesis and developing new therapeutics.

## Highlights

• PfSR10 constitutively couples with human Gi and Gq proteins
• Diverse antimalarial drugs directly activate PfSR10 signaling
• Key PfSR10 residues defined for antimalarial drug binding
• Host-derived bradykinin is identified as an endogenous agonist of PfSR10

## Graphical abstract

**Figure:**
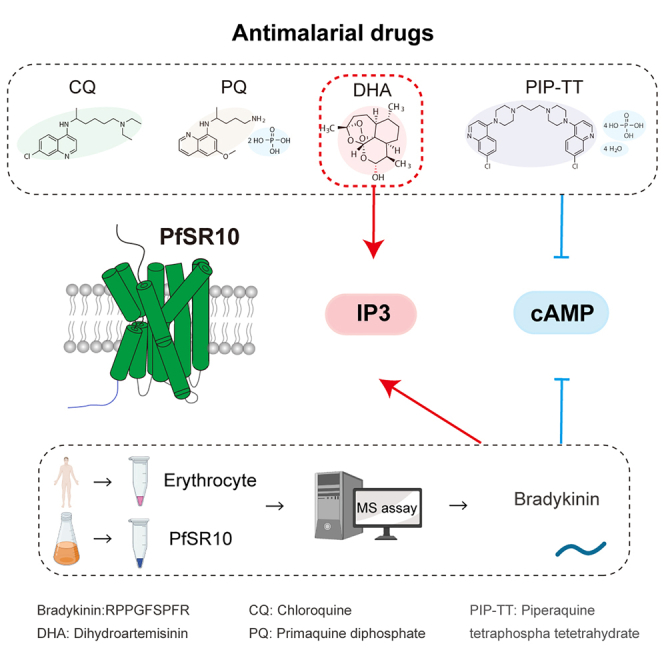

## Introduction

Treatment of virulent malaria-causing *Plasmodium* species infection remains a worldwide public health priority due to high mortality rates, especially in pregnant women and children, as well as the large contiguous populations exposed to infection.^1^^,^^2^^,^^3^^,^^4^^,^^5^^,^^6^^,^^7^ Although many antimalarial drugs have been developed during the past century, malaria-causing parasites have evolved drug resistance and remained potential threats to public health.^8^^,^^9^

Developing new antimalarial drugs requires knowledge of the mechanisms of both antimalarial drug resistance and key physiological processes governing the *Plasmodium* life cycle in hosts. However, our understanding of antimalarial drug actions and the signaling pathways underlying the parasite life cycle is incomplete. Notably, malaria-causing parasites are sophisticated single-cell organisms with a genome that encodes approximately 5,700 proteins. Approximately 31% of these 5,700 proteins are membrane proteins.^10^ Recent studies have suggested that *Plasmodium* membrane proteins not only modulate intraparasite signaling but can also be transferred to the plasma membrane of host erythrocytes, making them important antimalarial drug targets.^11^ Downstream of these membrane proteins, second messengers such as cAMP and calcium in the intracellular stores of both *Plasmodium* and host erythrocytic cells actively participate in the life cycle of malaria-causing parasites.^12^^,^^13^^,^^14^^,^^15^^,^^16^^,^^17^^,^^18^ In mammals, the intracellular concentrations of these second messengers are often regulated by G protein-coupled receptors (GPCRs), which constitute a superfamily of membrane receptors with seven transmembrane (TM) regions and serve as important drug targets.^19^^,^^20^^,^^21^^,^^22^^,^^23^ Intriguingly, several seven-TM serpentine receptors (SRs) have been recently found to be encoded by malaria-causing *Plasmodium* species.^14^ In particular, SR10, was identified as essential in the coordination of host rhythms with parasites in the intraerythrocytic developmental cycle (IDC), which is essential for the pathogenicity of malaria.^24^ However, key aspects of PfSR10 signaling and functions were still not fully elucidated, including the identity of PfSR10 ligand agonists, the ability of PfSR10 to sense host signals, and the possible connection between the parasite receptor and host G proteins and subsequent modulation of host intracellular second messengers.

## Results

### Constitutive activation and interaction of PfSR10 with G proteins

**Figure 1:**
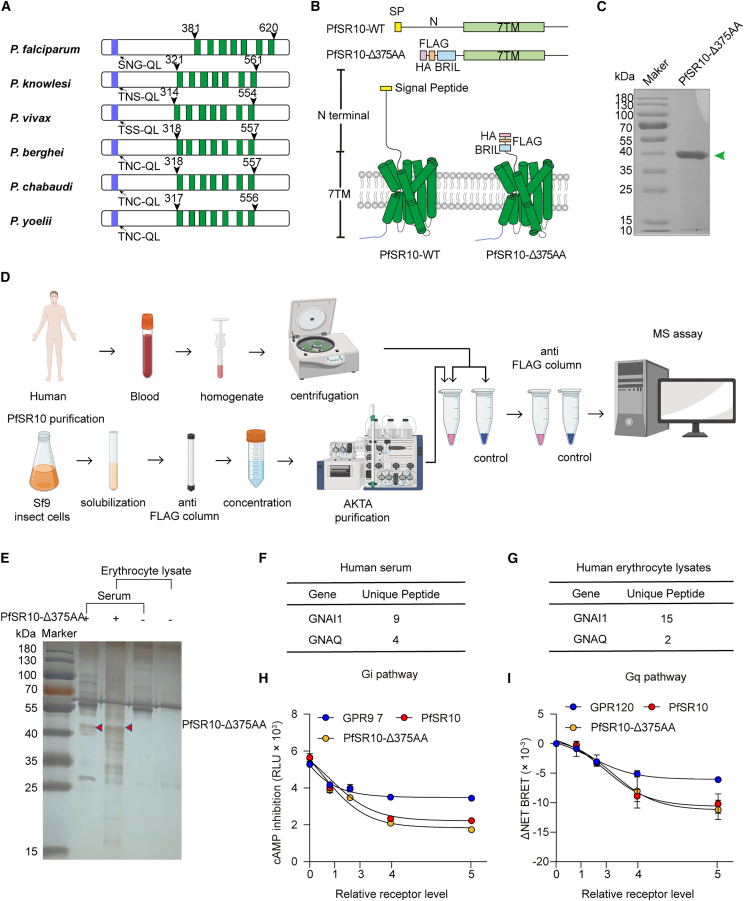
Constitutive activation and interaction of PfSR10 with G proteins (A) Diagram showing the architecture of SR10 in *P*.*falciparum*, *P*.*knowlesi*, *P*.*vivax*, *P*. *berghei*, *P*.*chabaudim*, *P*.*yoelii*. The putative seven-transmembrane helixes were shown in green. The signal peptides were shown in blue. And the signal peptide was computationally predicted using PlasmoDB. (B) Schematic presentation of the PfSR10 constructs used in this study, indicating the modifications and tags. Note that for expression in insect cells, the N-terminal region (1–375) of PfSR10 containing its original signal peptide (SP, yellow) was removed and a hemagglutinin (HA, purple) signal peptide followed by a FLAG tag (orange) and a BRIL epitope (cyan) fused at the N-terminus of PfSR10. (C) Representative coomassie-stained SDS-PAGE of the PfSR10 purified from insect cells. (D) Flowchart showing the identification of potential endogenous PfSR10 interacting proteins using mass spectrometry (MS)-based proteomic strategy. (E) Silver-staining analysis of the immunoprecipitated PfSR10 interacting proteins separated by SDS-PAGE. Note that the band (red arrow) of PfSR10 was observed at approximately 40 kDa. (F and G) Identification of human G protein α subunits GNAI and GNAQ as PfSR10 interacting proteins in human plasma (F) and erythrocyte lysate (G) by mass spectrometry analysis. (H and I) Constitutive activation of PfSR10, PfSR10-Δ375 and GPR97 Gi (H) or PfSR10, PfSR10-Δ375 and GPR120 Gq (I) signaling pathways determined by Glosensor-cAMP assay or G protein dissociation BRET assay. Data are represented as mean ± SEM from three independent experiments.

Four SR-like proteins have been identified in the human malaria-causing parasite *Plasmodium falciparum*: PfSR1, PfSR10, PfSR12, and PfSR25.^14^ The PlasmoDB search database (https://plasmodb.org/plasmo/app) lists six SR10 proteins in primate-infecting *Plasmodium* species (*P*. *falciparum*, *Plasmodium vivax*, and *Plasmodium knowlesi*) and rodent plasmodium (*Plasmodium chabaudi*, *Plasmodium berghei*, and *Plasmodium yoelii*), which share more than 70% homology. All SR10 proteins^25^ have a large N-terminal domain and a potential signal peptide. Among parasites with known SR10 sequences, *P*. *falciparum* is the deadliest human malaria-causing parasite (Figure 1A). Due to the pathological relevance of PfSR10, we performed proteomic analysis to identify the interaction partners of PfSR10 in human blood. We screened several PfSR10 constructs and found that the deletion of 375 amino acids in the N-terminus of the receptor significantly increased the receptor expression level (Figures 1B and 1C). We then introduced a hemagglutinin (HA) signal sequence followed by a FLAG tag in the PfSR10 N-terminus, expressed and purified the receptors from *Spodoptera frugiperda* (Sf9) cells (Figure 1D), and then incubated them with either a serum membrane fraction or human blood erythrocyte lysate. Anti-FLAG M1 beads were used to pull down proteins interacting with PfSR10. In addition, the membrane or lysate was incubated with anti-FLAG M1 beads to generate negative controls. The electrophoresis gel was silver stained to evaluate the efficiency of the PfSR10 pull-down assay (Figure 1E). Intriguingly, both Gq and Gi proteins were found to directly interact with PfSR10 (Figures 1F and 1G; Tables S4 and S7).

Notably, although PfSR10 contains seven TM regions and is thought to be a GPCR, PfSR10 G protein-coupling ability had not been previously examined, and its binding ligands had not been elucidated. Because recent studies have suggested that *Plasmodium* membrane proteins can be transferred to the erythrocyte plasma membrane,^25^^,^^26^ we suspected that PfSRs may couple to mammalian G proteins with or without ligand stimulation. We therefore sought to determine whether PfSR10 shows constitutive activity and regulates the level of the intracellular second messenger cAMP, a classical Gi/Go downstream. We utilized GPR97, a well-characterized Go-coupled receptor, as the control. PfSR10 and its truncated variant PfSR10-Δ375AA constitutively inhibited increase in intracellular cAMP level induced by forskolin (Fsk) in a concentration-dependent manner as determined on the basis of the amount of transfected plasmid (Figures 1H, S1A, and S1C). In addition to potential Gi/Go activity, PfSR10 and PfSR10-Δ375AA exhibited constitutive Gq-coupling activity as determined via Gq dissociation assay with GPR120 (a well-characterized Gq-coupled receptor) used as the control (Figures 1I and S1B). Compared to full-length PfSR10, PfSR10-Δ375AA exhibited equivalent constitutive activity for both Gi and Gq pathways.

### cAMP and G protein dissociation assay identification of four antimalarial drugs that are PfSR10 ligands

**Figure 2:**
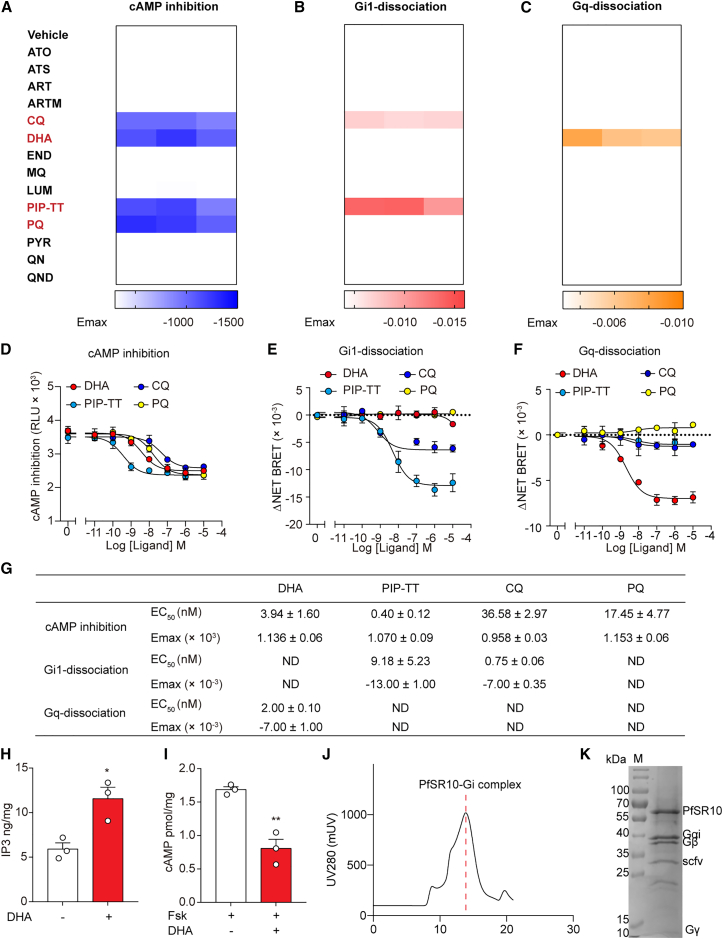
Identification of four antimalarial drugs as PfSR10 ligands using cAMP and G protein dissociation assays (A–C) Heatmap representing the cAMP inhibition (A), and Gi1 (B, left panel) or Gq (C, right panel) dissociation induced by anti-malaria drugs (10 μM) in HEK293 cells overexpressing PfSR10. DMSO at 0.1% concentration was used as the control (Vehicle). The blue and red color scales indicate the PfSR10 efficacy levels (Emax value) in inducing cAMP inhibition or activating Gi pathway using a Gi1-Gγ dissociation assay, respectively. Whereas the green color scale indicates the Gq pathway using a Gq-Gγ dissociation assay. The heatmap are generated according to the data shown in Figures S3A–S3F. Note that CQ, DHA,PIP-TT, and PQ remarkably activate Gi or Gq pathways in PfSR10 overexpressed HEK293 cells. Data were from three independent experiments (*n* = 3). (D–F) Representative concentration-dependent curves of CQ-, DHA-, PIP-TT-, and PQ-induced cAMP inhibition (D), Gi1 (E), or Gq (F) dissociation in PfSR10-overexpressing HEK293 cells. Data in (D-F) are shown as mean ± SEM of three independent experiments (*n* = 3). (G) Summarized potency and efficacy of CQ, DHA, PIP-TT, and PQ on HEK293 cells overexpressing PfSR10. Values are the mean ± SEM of three independent experiments, each performed in triplicate. The data are generated from Figures 2D–2F. (H) Effects of 10 μM DHA treatment on the IP3 concentration in isolated parasites at trophozoite stage. ∗*p* < 0.05, isolated parasites with 10 μM DHA treatment compared with untreated group. All the data were analyzed by Student’s *t* test. Data are shown as mean ± SEM of three independent experiments (*n* = 3). (I) Effects of 10 μM DHA treatment on the cAMP concentration in response to stimulation with 3 μM Fsk in isolated parasites at trophozoite stage. ∗∗*p* < 0.01, isolated parasites with 10 μM DHA treatment compared with untreated group. All the data were analyzed by Student’s *t* test. Data are shown as mean ± SEM of three independent experiments (*n* = 3). (J) Superose 6 size-exclusion chromatography profile of the PfSR10-Gi-scFv16 complex purified by FLAG-M1 affinity chromatography. (K) Coomassie-stained SDS-PAGE gel of the isolated gel-filtration peak fraction from Figure S4A.

We selected 15 clinical antimalarial drugs with diverse structural folds, and by measuring cAMP levels and performing Gi1/Gq protein dissociation assays, we sought to determine their ability to elicit PfSR10 activity. We used well-characterized GPCRs as positive controls: angiotensin II receptor (AT1R) and dopamine D2 receptor (D2R)^27^^,^^28^ (Figures 2A–2C, S2A, and S3J–S3L). Importantly, four antimalarial drugs, chloroquine (CQ), dihydroartemisinin (DHA), piperaquine-tetraphosphate-tetrahydrate (PIP-TT), and primaquine diphosphate (PQ), were found to inhibit Fsk-induced cAMP elevation via PfSR10 activation in a concentration-dependent manner (half maximal effective concentration [EC50] values were 36.58 ± 2.97 nM, 3.94 ± 1.60 nM, 0.40 ± 0.12 nM, and 17.45 ± 4.77 nM, respectively) (Figures 2A, 2D, 2G, S3A, S3B, and S3G). Both CQ and PIP-TT promoted Gi1 coupling with PfSR10 in a G protein dissociation assay (Figures 2B, 2E, 2G, S3C, S3D, and S3H). Moreover, DHA induced Gq dissociation at an EC50 of 2.00 ± 0.10 nM (Figures 2C, 2F, 2G, S3E, S3F, and S3I). In addition to human G proteins, previous studies have suspected G protein-second messenger systems also exist in *Plasmodium*.^12^^,^^13^ We therefore stimulate *Plasmodium* with anti-malarial drugs which were able to activate PfSR10, to see whether these drugs are connected to the second messenger systems of *Plasmodium*. We isolated *Plasmodium falciparum* in the trophozoite stage from infected red blood cells and examined the effects of DHA on IP3 concentration and Fsk-stimulated cAMP in *Plasmodium falciparum* cytoplasm. We found DHA significantly increased the concentration of IP3 and inhibited Fsk-Stimulated cAMP in the malaria parasite (Figures 2H and 2I). Future studies using PfSR10 knockout animals to identify potential G proteins in *Plasmodium* are required to determine the contribution of the PfSR10 in DHA-induced second messenger changes in *Plasmodium*.

### *In vitro* reconstitution of the PfSR10-Gi complex

To verify a direct interaction between PfSR10 and G proteins, we reconstituted the PfSR10-Gi trimer complex *in vitro* using a previously established procedure for complex formation of human GPCRs with corresponding G proteins^29^^,^^30^^,^^31^ (Figure S4A). Supplementation with the conformation-specific single-chain antibody scFv16, apyrase, and the antimalarial drug PIP-TT (final concentration: 10 μM) enabled the formation of a stable PfSR10:Gαi1:Gβ1γ2:scFv16 complex (Figures 2J and 2K). Most of the complexes assessed with gel filtration chromatography resembled monomers. In addition, single particle analysis was performed via electron microscopy (EM), and the 2-dimensional average clearly indicated the receptor region in micelles and the G protein region (Figures S4B–S4D; Table 1). These results confirmed the direct interaction between PfSR10 and the Gi1 trimer protein. In addition, the formation of the PfSR10-Gq-Gβ1γ2 complex was detected by western blotting (Figure S4E).

### PfSR10 conformational changes induced by antimalarial drugs

**Figure 3:**
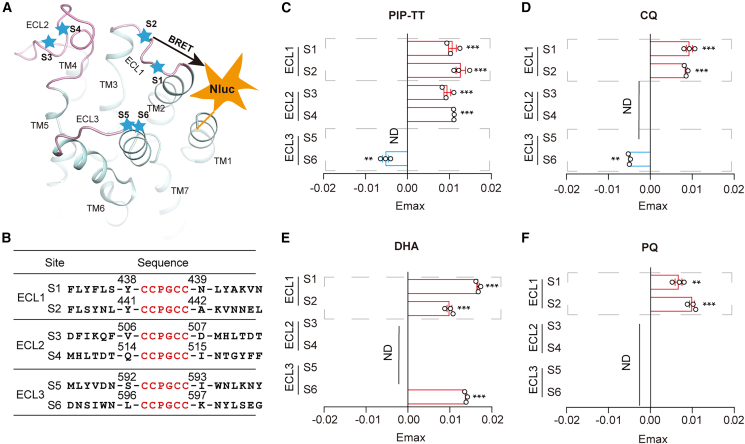
Conformational changes of PfSR10 induced by anti-malaria drugs (A) Cartoon representation of the FlAsH-BRET assay design based on the PfSR10 structural model generated using SWISS-MODEL. NanoLuc (Nluc) was inserted at the N-terminus of PfSR10, and the FlAsH motif (-CCPGCC-) was inserted at designated locations in the predicted extracellular loops (ECLs) of the PfSR10 as indicated by the blue pentagrams. (B) Detailed information of the FlAsH motif insertion sites at the extracellular loops of PfSR10 according to 3D structure shown in A. FlAsH motifs are highlighted in red. (C–F) The maximal responses of six PfSR10 FlAsH-BRET sensors in response to PIP-TT (C), CQ (D), DHA (E), and PQ (F) stimulation. ∗*p* < 0.05, ∗∗*p* < 0.01, ∗∗∗*p* < 0.001; ND, not detectable; FlAsH-BRET sensors stimulated with anti-malaria drugs were compared with those stimulated with control vehicle. All data were statistically analyzed using one-way ANOVA with Dunnett’s post hoc test. Data are presented as mean ± SEM from three independent experiments (*n* = 3).

To investigate ligand-induced PfSR10 conformational changes, we used the fluorescein arsenical hairpin-bioluminescence resonance energy transfer (FlAsH-BRET) method as previously described.^29^^,^^30^^,^^32^ We generated six FlAsH-BRET-based biosensors at “extracellular sites” according to a computationally stimulated PfSR10 model created by homology modeling with SWISS-MODEL (https://swissmodel.expasy.org/) (Figure 3A). Specifically, Nluc was fused to the PfSR10 N-terminus, and the “EDT2” binding sequence (-CCPGCC-) was inserted at six conformational biosensor sites within the predicted extracellular loop (ECL) regions (Figure 3B). The binding of PIP-TT, CQ, DHA, or PQ to PfSR10 caused inward movement of ECL1 toward the PfSR10 N-terminus in a concentration-dependent manner, with the largest BRET signal produced upon DHA stimulation (Figures 3C–3F and S5A–S5E). Moreover, separation of ECL3 from the PfSR10 N-terminus was observed only in the presence of PIP-TT and CQ, and it was observed at the S6 (site 6) sensor site with the FlAsH-labelled ECL3 motif (Figures 3C–3F and S5A–S5E). In addition, atovaquone (ATO) did not induce detectable extracellular conformational changes in PfSR10, ruling out the possibility of nonspecific conformational effects (Figure S5F). These results supported the supposition that PIP-TT, CQ, DHA, and PQ are PfSR10 ligands that can bind PfSR10 and induce different PfSR10 conformational changes.

### Molecular determinants of PfSR10 in the recognition of antimalarial drugs

**Figure 4:**
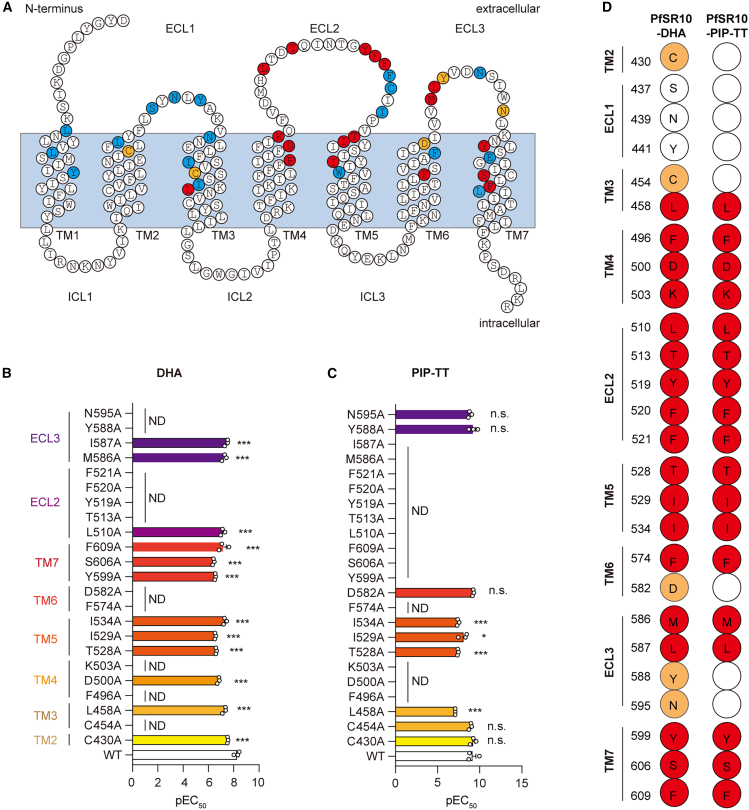
Binding model of DHA and PIP-TT with PfSR10 (A) Serpentine plot representation of PfSR10 residues of seven transmembrane region. Amino acid residues are colored according to the activity changes: Residues of which the mutation led to decreased potency for two ligands- (inducing cAMP inhibition) were colored in red, while residues of which the mutation led to decreased potency for DHA-stimulated cAMP inhibition were colored in orange, and residues of which the mutation had not impaired ligand-induced cAMP inhibition were colored in blue. (B and C) Alanine scanning identified important residues in PfSR10 receptor that recognize DHA (B), PIP-TT (C) through a Glosensor-cAMP assay. The EC50 values were calculated to characterize the coupling efficiency between PfSR10 and anti-malaria drugs. The pEC50 values are generated according to the data shown in Figure S6C. ∗∗*p* < 0.01, ∗∗∗*p* < 0.001, n.s., no significant difference, ND, not detectable; PfSR10 mutants were compared with wild-type PfSR10. All data were statistically analyzed using one-way ANOVA with Dunnett’s post hoc test. Data were from three independent experiments (*n* = 3). The bars indicate mean ± SEM values. (D) The residues in PfSR10 that contact with DHA, PIP-TT. Residues of which the mutation led to decreased potency for two ligands- (inducing cAMP inhibition) were colored in red, while residues of which the mutation led to decreased potency for DHA-stimulated cAMP inhibition were colored in orange.

To characterize the engagement of antimalarial drugs with PfSR10, we performed alanine (Ala) scanning mutagenesis of PfSR10 by altering all residues in the ECL and the upper half of the seven-TM bundle (7TM), which has side chains facing the intra-7TM bundle, according to SWISS-MODEL, and may constitute the ligand pocket^29^^,^^33^^,^^34^ (Figure 4A). After adjusting the amounts transfected, 41 PfSR10 mutants showed expression levels and cell surface localization similar to those of wild-type PfSR10, as determined by our ELISA results (Figures S6A and S6B). We therefore performed a concentration-dependent GloSensor-cAMP assay with these PfSR10 mutants. Importantly, mutations in 14 residues in the TM bundles and nine residues in the ECLs significantly impaired cAMP signaling downstream of PfSR10 in response to DHA stimulation (Figures 4B, 4D, and S6C). In total, 18 residues were identified as critical for PIP-TT-induced PfSR10 activation (Figures 4C and 4D). Notably, 11 mutations in TM3-TM7 and seven mutations in ECL2-ECL3 were identified as potential PfSR10-interacting residues shared by both antimalarial drugs DHA and PIP-TT (Figure 4D).

### Ligand selectivity mechanisms of SR10 toward DHA in divergent species

**Figure 5:**
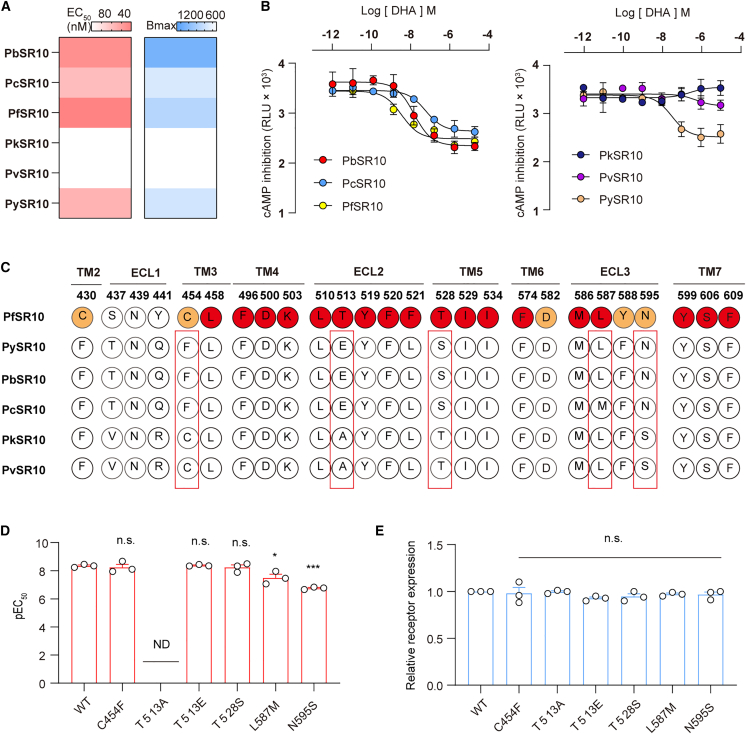
Ligand selectivity mechanisms of PfSR10 toward DHA in divergent species (A) Heatmap representing the cAMP inhibition by DHA in HEK293 cells overexpressing SR10 from different species. Heatmap shows the EC50 (left) and Bmax (right) values. The heatmap are generated base on the data shown Figue 5B. Data were from three independent experiments (*n* = 3). (B) Representative concentration-dependent curves of DHA-stimulated cAMP inhibition in HEK293 cells with overexpressing different species SR10. Data are represented as mean ± SEM of three independent experiments. (C) Conservation analysis of functionally critical residues in SR10 orthologs from diverse Plasmodium species. (D) Bar graph showing the pEC50 of DHA in the activation of wild-type (WT) or mutant PfSR10. Data are represented as mean ± SEM of three independent experiments. Statistical differences between WT PfSR10 and its mutants were determined by one-way ANOVA, with significance levels indicated as ∗*p* < 0.05, ∗∗*p* < 0.01, ∗∗*p* < 0.001. (E) Relative expression levels of wild type PfSR10 and alanine mutants of putative binding sites at seven transmembrane domains and three extracellular loops were measured by ELISA assay. Statistical differences between WT PfSR10 and its mutants were determined by one-way ANOVA, with significance levels indicated as n.s., no significant difference. Data are represented as mean ± SEM of three independent experiments.

Subsequently, using GloSensor-cAMP assay, we examined the activation of SR10 by DHA in different species. SR10, derived from six different *Plasmodium* species, was expressed at similar levels in HEK293 cells, as confirmed by ELISAs (Figure S7H). Notably, DHA was found to inhibit Fsk-induced cAMP elevation via PbSR10, PcSR10, and PySR10 activation in a concentration-dependent manner with EC50 values of 12.28 ± 4.75 nM, 37.58 ± 15.49 nM, and 36.59 ± 6.78 nM, respectively. But the two other *Plasmodium* species showed no response to DHA (Figures 5A and 5B). To elucidate the molecular basis for species-selective DHA agonism, we aligned SR10 ortholog sequences and evaluated conservation of residues critical for ligand binding in PfSR10 (Figure 5C). Five positions exhibited notable divergence across *Plasmodium* species: C454, T513, T528, L587, and N595 (Figure 5C). Site-directed mutagenesis of PfSR10 revealed that three substitutions—T513A, L587M, and N595S—significantly impaired DHA-induced cAMP inhibition, while C454F and T528S mutations showed minimal effects on DHA potency (Figures 5D and 5E). These findings identify T513, L587, and N595 as evolutionarily variable residues governing species-selective DHA sensitivity in *Plasmodium* SR10 receptors.

Additionally, using Gi protein trimer dissociation assays, we examined the activation of SR10 by these four antimalarial drugs in different species. Notably, DHA and PQ promoted Gi dissociation downstream of PbSR10 in *P*. *berghei* (a rodent-infecting *Plasmodium* species), but the four other *Plasmodium* species showed no response to these antimalarial drugs (Figures S7A–S7G).

### Identification of bradykinin and des-Arg9-bradykinin as PfSR10 ligands

**Figure 6:**
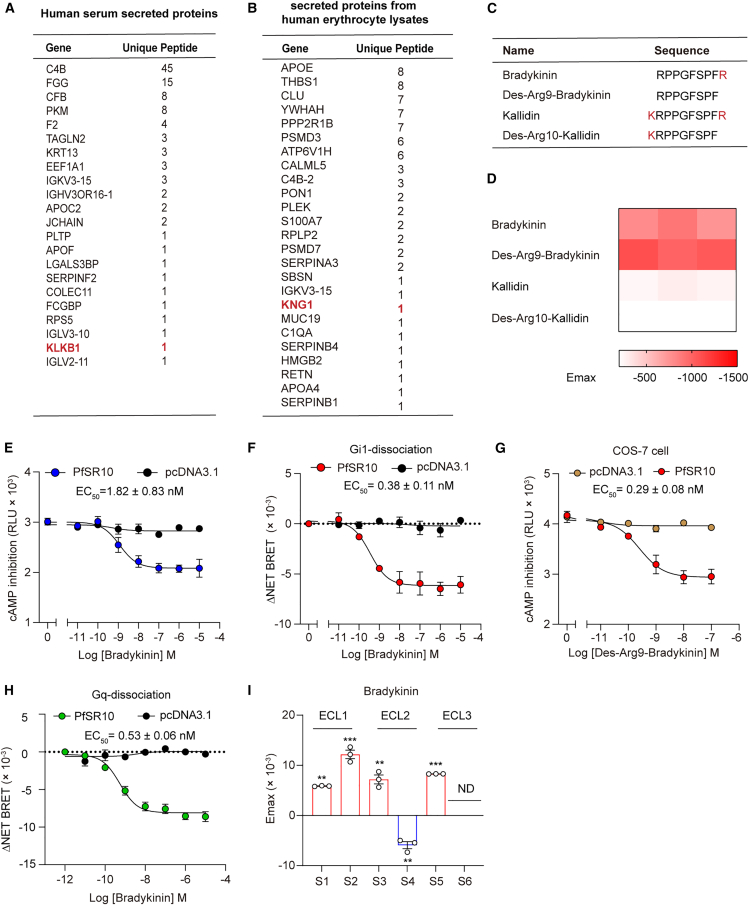
Identification of bradykinin as ligand of PfSR10 (A and B) Secretory proteins from human serum (A) and erythrocyte (B) potentially interacting with PfSR10 identified by mass spectrometry-based proteomic analysis. (C) The amino acid sequence of bradykinin (RPPGFSPFR), des-Arg9-bradykinin (RPPGFSPF), kallidin (KRPPGFSPFR), and Des-Arg10-kallidin (KRPPGFSPF). (D) Heatmap representing the cAMP inhibition induced by various peptides (10 μM) in HEK293 cells or COS-7 cells overexpressing PfSR10. Data were from three independent experiments (*n* = 3). (E–H) Representative concentration-dependent curves of bradykinin or des-Arg9-bradykinin-induced cAMP inhibition (E, H), Gi (F) and Gq (G) dissociations in PfSR10-overexpressing HEK293 cells or COS-7 cells.Data are shown as mean ± SEM from three independent experiments (*n* = 3). (I) The maximal responses of six PfSR10 FlAsH-BRET sensors in response to bradykinin stimulation. ∗∗*p* < 0.01, ∗∗∗*p* < 0.001; ND, not detectable; FlAsH-BRET sensors stimulated with bradykinin was compared with stimulated with control vehicle. All data were statistically analyzed using one-way ANOVA with Dunnett’s post hoc test. Data are shown as mean ± SEM from three independent experiments (*n* = 3).

The PfSR10 protein interactome of human blood erythrocytes provided us with an opportunity to identify potential PfSR10 ligands in the human body. We determined positive hits from proteins listed as potential PfSR10 interactors by selecting candidates present in only the PfSR10 group or with a peptide intensity in the PfSR10 group that was at least 1.5-fold higher than that in the control group. We identified a total of 82 and 243 putative PfSR10-interacting proteins from human serum and erythrocyte lysate, respectively, including 71 secretory proteins that may be present in the extracellular environment (Figures 6A and 6B; Tables S2–S7). In particular, we noticed that the list included kallikrein and kininogen-1 in the human erythrocyte plasma membrane fraction and erythrocyte lysate, respectively; these proteins are key components of the kallikrein-kinin system (KKS). The KKS plays essential roles in regulating blood pressure and skin wound healing and is associated with renal disease and pain management.^35^^,^^36^^,^^37^ The main KKS components include four biologically active natural kinin peptides, bradykinin (BK, RPPGFSPFR), kallidin (Lys-bradykinin, Lys-BK, KRPPGFSPFR), and their carboxy-terminal des-Arg metabolites(des-Arg9-bradykinin, des-Arg9-BK, RPPGFSPF and des-Arg10-kallidin, Lys-des-Arg9-BK, KRPPGFSPF, respectively), derived from different kininogen isoforms.^38^ To determine whether the KSS system regulated PfSR10 activity, we stimulated PfSR10-overexpressing HEK293 cells with four kinin peptides (Figures 6A–6D, S8A, and S8B). Importantly, bradykinin and des-Arg9-bradykinin significantly inhibited Fsk-induced cAMP level elevation and promoted Gi1 coupling to PfSR10 in a concentration-dependent manner (Figures 6E and 6F). We used COS-7 cells to examine the activation of PfSR10 in response to des-Arg9-bradykinin stimulation (Figure 6G). Furthermore, bradykinin stimulation led to a remarkable reduction in the Gq dissociation signal (Figure 6H). Assessment of the FlAsH-BRET sensors indicated movement of ECL1 and ECL3 toward the N-terminus and separation of the ECL2 region and N-terminus of PfSR10 in response to bradykinin or des-Arg9-bradykinin engagement (Figures 6I and S8C–S8E). Collectively, these results suggest that bradykinin and des-Arg9-bradykinin are potent ligands of PfSR10.

### Molecular basis of bradykinin recognition and species-specific activation of SR10

Through alanine-scanning mutagenesis of potential ligand-binding residues, we identified 19 critical sites governing bradykinin-induced PfSR10 activation (Figures S9A and S9B). Among these, 14 residues (eight in TM3-TM7 domains and six in ECL2-ECL3 regions) represent shared interaction sites with antimalarial drugs (Figure S9A). All mutants retained wild-type expression levels (Figures S6A and S6B). Finally, bradykinin promoted Gi activity downstream of PcSR10 and PySR10 in a concentration-dependent manner with EC50 values of 0.15 ± 0.005 nM and 0.67 ± 0.29 nM, respectively (Figures S9C–S9E).

## Discussion

Although the function of PfSR10 in *P*. *falciparum* has not been elucidated to date, recent studies have shown that SR10 regulates the IDC by coordinating host rhythms and malaria-causing parasites.^24^ However, PfSR10 ligands were unknown, and the downstream signaling effectors were unclear. In this study, by performing second messenger, G protein dissociation and FlAsH-BRET-based conformational change assays, we identified CQ, DHA, PIP-TT, PQ, and bradykinin as PfSR10 ligands. Therefore, determining whether the SR10 ligands identified herein, including four antimalarial drugs and bradykinin, can modulate the IDC of malaria-causing *Plasmodium* and thus contribute to antimalarial effects by activating PfSR10 remains an intriguing area of inquiry.

Another important unaddressed issue is whether there is a receptor-G protein signal transduction system in *Plasmodium* that parallels that in mammals. Interestingly, recent studies have reported that effectors downstream of G proteins, such as PKA, PDE, and PLC, are present in the *Plasmodium*.^12^^,^^13^^,^^16^^,^^18^ Our finding indicating an association of SR10 with human G proteins suggests that effectors similar to G proteins may exist in *Plasmodium*, supporting the aforementioned hypothesis.^39^ In particular, we have identified that one of the malaria drugs which was able to bind and activate PfSR10, the DHA, was able to modulate intracellular IP3 and cAMP level in *Plasmodium*. Future studies based on proteomics combined with agonist-PfSR10 complex pull-down assays to discover downstream effectors are in urgent need in the identification of potential G proteins in *Plasmodium*. Notably, it has been reported that hundreds of membrane proteins can be transported to the plasma membrane of erythrocytes by *Plasmodium* to ensure rapid parasite reproduction. Therefore, producing an antibody that specifically recognizes PfSR10 or engineering a tag to label PfSR10 in *Plasmodium* may help clarify whether PfSR10 can be transferred to the membrane of erythrocytes and engage with human G proteins in response to agonist stimulation. On the basis of our 2-dimensional cryo-EM results, optimization of the PfSR10-Gi complex reconstitution procedure for cryo-EM structure resolution may facilitate our understanding of SR10-sensing human ligands or antimalarial drugs. Taken together, our experiments enabled us to identify antimalarial drugs, including CQ, DHA, PIP-TT, PQ, bradykinin, and des-Arg9-bradykinin, which are ligands of PfSR10. Notably, although *PfSR10* knockout is tolerated *in vitro*,^24^ our data indicate it functions as a signaling integrator for both host-derived cues (e.g., bradykinin) and antimalarial compounds (e.g., DHA). This dual-ligand sensing capability (not previously documented in other *Plasmodium* receptors) may permit functional compensation during *in vitro* culture. The observed knockout tolerance could reflect stage-specific roles, consistent with rapid DHA-induced IP_3_ elevation in trophozoites. These findings suggest therapeutic potential: PfSR10 targeting might simultaneously disrupt parasite messengers (cAMP/IP_3_) and host-parasite crosstalk, offering multi-pathway intervention opportunities. Moreover, bradykinin has also been identified as a ligand of both PcSR10 and PySR10. Interestingly, engagement of these ligands with SR10 induced downstream G protein coupling, suggesting that SR10 may be a GPCR. Future studies using modeled and structural solutions of the *Plasmodium* SR10 in complex with these ligands or G proteins may greatly help delineate the biochemical and functional details of SR10 in different pathophysiological processes.

### Limitations of the study

Although our study identifies PfSR10 as a membrane receptor for multiple antimalarial drugs and reveals its coupling to human G proteins, several limitations remain. First, we were unable to resolve the final high-resolution cryo-EM structure of the PfSR10-ligand-G protein complex, potentially due to the use of human G proteins rather than the native *Plasmodium* G proteins. Future structural studies using endogenous *Plasmodium* G proteins may facilitate successful determination of the complex architecture and elucidate precise mechanisms of drug recognition. Second, although we biochemically identified several antimalarial drugs as PfSR10 agonists, functional validation using *Plasmodium* SR10 knockout parasites has not been performed. Future studies involving genetic ablation of SR10 in *Plasmodium* will be essential to confirm the physiological relevance of PfSR10 in drug action and parasite biology.

## Resource availability

### Lead contact

Further information and requests for resources should be directed to and will be fulfilled by the lead contact, Jin-Peng Sun (sunjinpeng@bjmu.edu.cn).

### Materials availability

This study did not generate new unique reagents.

### Data and code availability

• All data supporting the findings of this study are available within the paper and its supplemental information.
• This paper does not report original code. Any additional information required to reanalyze the data reported in this paper is available from the lead contact upon request.

## Acknowledgments

We thank the Cryo-EM Center at the Biomedical Research Center for Structural Analysis of Shandong University for the support in cryo-EM data collection. We thank the Translational Medicine Core Facility at the Advanced Medical Research Institute, Shandong University, for their technical support. This work was supported by 10.13039/501100001809National Natural Science Foundation of China (81773704 to J.-P.S., 92057121 to X.Y., and 82304583 to H.L.); 10.13039/501100014219National Science Fund for Distinguished Young Scholars grant (82425105 to J.-P.S. and 82225011 to X.Y.); State Key Program of National Natural Science Foundation of China (82330118 to J.-P.S.). Noncommunicable Chronic Diseases-National Science and Technology Major Project (2024ZD0523100). National Key R&D Program of China (2024YFA0916900 to J.-P.S). Noncommunicable Chronic Diseases-National Science and Technology Major Project (2023ZD0506900 to X.Y.). Noncommunicable Chronic Diseases-National Science and Technology Major Project (2023ZD0506900 to X.Y.). National Natural Science Foundation of Shandong Province (ZR2023QH189 to H. L.); Open Research Project in State Key Laboratory of Vascular Homeostasis and Remodeling (Peking University); National Natural Science Foundation of China (32371363 to P.Z.); 10.13039/501100007129Natural Science Foundation of Shandong Province (ZR202211150041 to P.Z.);10.13039/100014717National Science Fund for Excellent Young Scholars (32222038 to P.X.); Shandong Provincial Natural Science Fund for Distinguished Young Scholars (ZR2024JQ013 to P.X.); and Taishan Scholars Program NO.tsqn202211015.

## Author contributions

J.-P.S. and X.Y. conceived the overall project. J.-P.S. conceived the idea that 7transmembrane receptor in Plasmodium may be tranferrable to mammalian cell membrane andinitiate G proteins signaling. J.-P.S., X.Y., H.L., P.X, and P.-J.Z designed and supervised all the experiments. J.-P.S., X.Y., P.X., P.-J.Z., X.W., and H.L. participated in data analysis and interpretation. D.J., Q.-X.C., and H.L. performed antimalarial drugs screening assay for PfSR10. D.J. and H.L. performed identification of endogenous ligands for PfSR10. P.X., X.W., and R.Z. generated the PfSR10 insect cell expression construct, established the PfSR10-Gi complex formation strategy, and prepared protein samples for cryo-EM. P.X., X.W., and R.Z. generated viruses and infected sf9 cells for large quantitative protein production. X.W. and R.Z. prepared negative-staining and evaluated the sample by negative-stain EM and prepared the cryo-EM grids, collected the cryo-EM data, and performed cryo-EM map calculation under the supervision of P.X. J.-P.S., X.Y., D.J., and H.L. designed all the mutations for determination of ligand binding sites. H.L. and D.J. generated all constructs and mutants for the cell-based functional assays. H.L., D.J., and Q.-X.C. performed ELISA assays, G protein dissociation assays, and cAMP inhibition assays. D.J. performed FlAsH-BRET assays. B.H., X.Y., and J.-P.S. designed the *Plasmodium* functional experiments. H.L. and F.Y. performed analysis of biological information. H.L. and K.Z. performed functional studies. H.L., P.X., F.Y., and J.-F.H. participated in functional assay design and prepared figures. J.-P.S., X.Y., and P.X. wrote the manuscript with input from all authors.

## Declaration of interests

The authors declare no competing interests.

## STAR★Methods

### Key resources table

**Table:** 

REAGENT or RESOURCE	SOURCE	IDENTIFIER
**Antibodies**		

Anti-FLAG monoclonal antibody (M2)	Sigma-Aldrich	Cat# F1804; RRID: AB_262044
Anti-Gαq polyclonal antibody	Proteintech	Cat# 27264-1-AP; RRID: AB_2880822
Anti-Gβ polyclonal antibody	Proteintech	Cat# 10247-2-AP; RRID: AB_2111820
Anti-Gγ polyclonal antibody	Proteintech	Cat# 11693-1-AP; RRID: AB_2263277
HRP-conjugated goat anti-rabbit IgG	Sigma-Aldrich	Cat# A6154; RRID: AB_258284
HRP-conjugated goat anti-mouse IgG	Sigma-Aldrich	Cat# A0216; RRID: AB_2860575

**Bacterial and virus strains**		

Trans5α Chemically Competent Cell	TRANS	Cat# CD201
DH10Bac competent cell	Biomed	Cat# BC112-01

**Chemicals, peptides, and recombinant proteins**		

Chloroquine (CQ)	Topscience	Cat# T8689
Dihydroartemisinin (DHA)	MCE	Cat# HY-N0176
Piperaquine tetraphosphate tetrahydrate (PIP-TT)	Topscience	Cat# T7744
Primaquine diphosphate (PQ)	Topscience	Cat# T0850
Bradykinin (BK)	MCE	HY-P0206
des-Arg9-Bradykinin	ChinaPeptides	Custom synthesis
Kallidin	ChinaPeptides	Custom synthesis
des-Arg10-Kallidin	ChinaPeptides	Custom synthesis
Melatonin	MCE	Cat# HY-B0075
Coelenterazine-h	Promega	Cat# S2011
Coelenterazine 400a	Maokangbio.	Cat# MX4610
FlAsH-EDT2	Thermo Fisher	Cat# T34561
2xClonExpression Mix	Vazyme	Cat# C115-02-AA
GloSensor cAMP reagent	Progema	E1290
Trypsin-EDTA (0.25%)	Gibco	25200072
DMEM(Dulbecco’s Modified Eagle Medium)	Gibco	11995065
ESF 921 Insect Cell Culture Medium	ExpressionSystems	Cat# 96-001-01
Transfection Medium	ExpressionSystems	Cat# 95-020-100
FuGENE HD Tranfection Reagent	Promega	Cat# E2311
Lauryl Maltose Neopentyl Glycol (LMNG)	Anatrace	Cat# NG310
Cholesteryl HemisuccinateTRIS Salt (CHS)	Anatrace	Cat# CH210
Glyco-diosgenin (GDN)	Anatrace	Cat# GDN101
Apyrase	Sigma-Aldrich	Cat# A6535
Leupeptin	Sangon	Cat# A600580
Benzamidine hydrochloride	aladdin	Cat# B100967

**Critical commercial assays**		

cAMP ELISA Kit	R&D Systems	Cat# KGE012B
IP3 ELISA Kit	Cloud-Clone	Cat# CEC037Ge
Fast Silver Staining Kit	Beyotime	Cat# P0017S

**Deposited data**		

Cryo-EM map of PfSR10-Gi-scFv16 complex	This paper	N/A

**Experimental models: cell lines**		

HEK293	ATCC	Cat# CRL-1573
COS-7	ATCC	Cat# CRL-1651
*Spodoptera frugiperda* Sf9 cells	Expression Systems	Cat# 94-001F

**Experimental models: organisms/strains**		

*Plasmodium falciparum* 3D7	BEI Resources	MRA-102

**Oligonucleotides**		

PfSR10 mutagenesis primers	This paper	N/A

**Recombinant DNA**		

pcDNA3.1-PfSR10-FLAG	This paper	N/A
pFastBac1-PfSR10-Δ375-BRIL-FLAG	This paper	N/A
pFastBac1-DNGαi1	This paper	N/A
pFastBac1-scFv16	This paper	N/A
pFastBacDual-Gβ1-His-Gγ2	This paper	N/A

**Software and algorithms**		

GraphPad Prism 8	GraphPad Software	https://www.graphpad.com
SWISS-MODEL	Swiss Institute of Bioinformatics	https://swissmodel.expasy.org/
Proteome Discoverer 1.4	Thermo Scientific	https://www.thermofisher.com

**Other**		

Superose 6 Increase 10/300 GL	GE Healthcare	GE-29-0915-96
Quantifoil R1.2/1.3 300-mesh gold grids	Quantifoil	Q59042
6-well Cell Culture Plate	Lab Serv	Cat# No.310109006
96-well Cell Culture Plate	Lab Serv	Cat# No.310109027
Multimode Plate Reader	EnVision	Envision HTS

### Experimental model and study participant details

#### Cell lines

Human Embryonic Kidney 293 (HEK293) cells and African Green Monkey Kidney Cells (COS-7) cells were obtained from American Type Culture Collection (ATCC, Manassas, VA, USA) and cultured in DMEM with 10% FBS. Sf9 cells were purchased from Expression Systems (Cat 94-001S) and cultured according to the manufacturer’s instructions.

#### Parasites

*Plasmodium falciparum* strain 3D7 parasites were cultured in flasks at 37°C and 5% haematocrit in RPMI 1640 medium supplemented with 10% human plasma, gassed with 90% N_2_, 5% O_2_ and 5% CO_2_.^40^

#### Ethical statement

Human blood samples were obtained from Qilu Hospital of Shandong University. All experiments were approved by the Ethics Committee of Shandong University [Approval No. ECSBMSSDU2024-1-006].

### Method details

#### Regents

FLAG peptide (DYKDDDDK), bradykinin (BK, RPPGFSPFR), bradykinin-Lys-FITC (RPPGFSPFRK-FITC), FITC-bradykinin (FITC-RPPGFSPFR), kallidin (Lys-bradykinin, Lys-BK, KRPPGFSPFR) and its carboxy-terminal des-Arg metabolites des-Arg9-bradykinin (des-Arg9-BK, RPPGFSPF) and des-Arg10-kallidin (KRPPGFSPF) were synthesized and purified to >95% purity at China peptides Co., Ltd. (Shanghai, China). Artemisinin (ART) and Dihydroartemisinin (DHA) were purchased from Med Chem Express, and Atovaquone (ATO), Lumefantrine (LUM), Mefloquine hydrochloride (MQ), Piperaquine tetraphosphate tetrahydrate (PIP-TT), Primaquine diphosphate (PQ), Pyrimethamine (PYR), Chloroquine (CQ), Quinine (QN), Artemether (ARTM), Artesunate (ATS), Euquinine (EQN), and Quinidine (QND) were purchased from Topscience.

#### Constructs

The full-length cDNA sequence of *Plasmodium falciparum* serpentine receptor 10 (PfSR10, PlasmoDB: PF3D7_1215900) was synthesized at Tsingke Biological Technology Co., Ltd. (Beijing, China). To produce protein in insect cells, the cDNA encoding for *Plasmodium falciparum* serpentine receptor 10 (residues 376-655) was cloned into a modified pFastBac1 baculovirus expression vector with a hemagglutinin (HA) signal peptide followed by a FLAG tag (DYKDDDDA) and a thermostabilized apocytochrome b_562_ RIL (BRIL) epitope fused at the N-terminus of PfSR10.^41^ Dominant-negative human αi1 (DNGαi1, S47N, G203A, E245A, A326S) and GqiN (N-terminus (residue 1-32) of human Gαq was replaced with the N-terminus of Gi (residue 1–28)), and the single chain antibody scFv16 were cloned into pFastBac1,^31^ respectively. The human Gβ1 with C-terminal hexa-histidine tag and human Gγ2 were cloned into the pFastBacDual vector.^29^^,^^31^ In order to examine the activity of PfSR10, PfSR10 were cloned into the pcDNA3.1 vector with its N-terminus fused with hemagglutinin (HA) signal peptide followed by a FLAG tag (DYKDDDDK). PfSR10 mutants L382A, L387A, Y392A, C430A, L433A, S436A, N438A, Y440A, N445A, I451A, C454A, I457A, L458A, F496A, D500A, K503A, L510A, T513A, Y519A, F520A, F521A, F522A, C523A, I525A, T528A, F579A, D582A, M586A, I587A, Y588A, N591A, N595A, Y599A, E602A, S606A, F609A, L610A were generated by Quikchange mutagenesis kit (Stratagene). For determination of the extracellular domain conformational changes, etogether with a four-amino acid linker (Gly-Ser-Ser-Gly) were fused to the N-terminus of PfSR10 (at 382 position) and the FlAsH sequence (Cys-Cys-pro-Gly-cys-Cys) were inserted into designated regions in ECL1, ELC2 or ECL3 of NLUC-PfSR10, as shown in Figure 4B. All of the constructs were verified by DNA sequencing at Tsingke Biological Technology Co., Ltd. (Beijing, China).

#### Protein expression in insect cells

High-titer recombinant baculovirus was generated using the Bac-to-Bac Baculovirus Expression System (Invitrogen) as previously described.^42^ Briefly, *Spodoptera frugiperda* (Sf9) insect cells grown in ESF921 medium (Expression System) at a density of 2.5 × 10^6^ cells per mL were infected with ∼10 μg of PfSR10, DNGαi1, Gβ1γ2 or scFv16 recombinant bacmid using FuGENE HD transfection reagent (Promega). For protein expression, the Sf9 insect cells were infected at a density of 2.5 × 10^6^ cells per mL with PfSR10 baculovirus at a multiplicity of infection (MOI) ratio of 5.0, or coinfected with PfSR10, DNGαi1, Gβ1γ2 and scFv16 baculovirus at a multiplicity of infection (MOI) ratio of 1:2:1:1. Cells were harvested by centrifugation 48 h post-infection, flash-frozen in liquid nitrogen and stored at -80°C for further use.

#### PfSR10-Gi complexes formation and purification

The PfSR10-Gi-scFv16 complex was formed on membrane as previously described. Briefly, Sf9 cell pellets infected with PfSR10, Giheterotrimerand scFv16 were thawed on ice and lysed by resuspension in buffer containing 20 mM HEPES pH 7.5, 100 mM NaCl, 10 mM CaCl_2_, 5 mM MgCl_2_, 2.5 mg/mL leupeptin, 0.2 mg/mL benzamidine followed by homogenization. The complex was assembled on membrane by adding 10 μM of PIP-TT and 25 mU/mL apyrase, and further incubated at room temperature for 2 h with end-to-end rotation. Afterward, the complex was extracted from the cell membrane using 0.5% (w/v) lauryl maltose neopentylglycol (LMNG) (Anatrace) and 0.1% (w/v) cholesteryl hemisuccinate (CHS) (Anatrace) at 4°C for 2 h. Insoluble matter were removed by centrifugation at 25,000 rpm for 30 min, and the supernatant containing solubilized complex was further incubated with anti-Flag M1 affinity resin at 4°C for 2 h. The resin was then packed into a glass column, and washed stepwise with buffer (20 mM HEPES pH 7.5, 100 mM NaCl, 5 mM CaCl_2_, 2 mM MgCl_2_, 0.001% (w/v) CHS and 10 μM of piperaquine phosphate) containing decreasing concentrations of LMNG, from 0.1% to 0.002% (w/v) LMNG. The complex was eluted with 20 mM HEPES pH 7.4, 100 mM NaCl, 2 mM MgCl_2_, 0.01% (w/v) LMNG, 0.001% (w/v) CHS, 5 mM EGTA and 0.2 mg/mL FLAG peptide. The eluate was concentrated with a 100-kDa molecular weight cut-off (MWCO) Amicon Ultra centrifugal filter (Millipore) and subjected to size-exclusion chromatography using Superpose 6 10/300 increase column which was pre-equilibrated with 20 mM HEPES pH 7.4, 100 mM NaCl, 2 mM MgCl_2_, 0.01% (w/v) LMNG, 0.001% (w/v) CHS and 10 μM of PIP-TT. The eluted fractions consisting of PfSR10-Gi-scFv16 monomeric complex were pooled and concentrated to 15 mg/mL using a 100-kDa MWCO centrifugal filter for further preparation of cryo-EM grids.

#### Cryo-EM data collection and processing

For vitrification, 3 μL of the purified PfSR10-Gi-scFv16 complex sample at a concentration of approximately 15 mg/mL was applied to glow-discharged 300-mesh Au grids (Quantifoil,R1.2/1.3), and subsequently plunge-frozen in liquid ethane using a FEI Vitrobot Mark IV (Thermo Fisher Scientific) set at 4°C and 100% humidity. For data collection, cryo-EM imaging was performed on a Titan Krios microscope (Thermo Fisher Scientific) equipped with a K2 direct electron detector (Gatan) in counting mode, operated at 300 kV with the super-resolution pixel size at 1.08 Å. All movie stacks were collected with the serialEM software^43^ at an exposure rate of 60 electrons per Å^2^ per s (total exposure time 8 s/ 32 frames per movie stack) with defocus ranging from -1.0 to -2.0 μm. A total number of 4418 movies were collected, and the statistics of data collection are listed in Extended Data Table 1. Cryo-EM data processing was performed using RELION-3.1.0.^44^ A total of 3,626,256 particles for a cascade of 2D classification were picked from 4,418 usable micrographs of the PfSR10-Gi-scFv16 sample (The flowchart for processing vitrified PfSR10-Gi-scFv16 complexes were shown in Extended Data Figure 3B). All the collected micrographs were motion-corrected and dose-weighted by MotionCor2,^45^ contrast transfer function (CTF) parameters were estimated by GCTF.^46^

#### Co-immunoprecipitation and western blot analysis

To verify the direct interaction between PfSR10 and the Gq trimer protein. HEK293 cells were transfected with PfSR10 with Flag fused at N terminus, Gβ and Gγ. Forty-eight hours after transfection, the cells were washed in ice-cold HBSS buffer and then centrifuged at 600g for 5 min at 4°C. In brief, the whole cell pellet was solubilized with HBSS buffer containing protease inhibitor cocktail (Roche, 11697498001) and 1% DDM for 2 h at 4°C with end-to-end rotation, and further centrifuged at 60,000g for 30 min. Anti-Flag antibody covalently linked sepharose beads (Abcam, ab69314) were used to pull-down PfSR10. The cell lysates were immunoprecipitated using anti-Flag antibody sepharose beads at 4°C for 3 h with end-to-end rotation. The immunocomplexes were collected by centrifugation at 1,000g and washed four times with HBSS buffer in the presence (for cell plasma membrane samples) or absence of 1% DDM. After removing the supernatant, the samples were subjected to SDS–PAGE and western blot for further analysis. Immuno-staining against PfSR10, Gq, Gβ and Gγ were performed using anti-FLAG (Sigma, F1804), anti-Gq (Proteintech, #27264-1-AP), anti-Gβ (Proteintech, #10247-2-AP), and anti-Gγ (Proteintech, #11693-1-AP) specific antibodies with 1:1000 dilution, respectively. The HRP-conjugated goat anti-rabbit IgG or HRP-conjugated goat anti-mouse IgG were used as secondary antibodies at 1:2500 dilution.

#### ELISA assay

To compare the effect of wild-type PfSR10 or its mutants in the functional Gi/Gq protein dissociation essay and Glosensor-cAMP assays, we performed ELISA assay to determine their surface expression. HEK293 cells were cultured in a six-well plate and transfected with the target wild-type PfSR10 or its mutants. Transfected cells were then divided into 24 well plates at a cell density of 1×10^5^ and incubated at 37 °C and 5% CO_2_ concentration for 16 h. The cells were then immobilized with 4% (W/V) paraformaldehyde at room temperature for 15 min and sealed with 5% (W/V) bovine serum albumin at room temperature for 1 h. The cells were washed and incubated with monoclonal antibody Flag primary antibody (Catalog #F1804, Sigma Aldrich) at 4°C for 14h, followed by goat secondary antibody mouse antibody (A-21235, Thermo Fisher) at room temperature for 1h. After washing, TMB (3,3 ′, 5,5 ′-tetramethylbenzidine) solution was added and colour reaction was carried out under dark condition. An equal amount of 0.25m hydrochloric acid was added to quench reaction. Finally, the optical density at 450nm was measured using a TECAN luminescent counter (Infinite M200 Pro NanoQuant) and plotted against the transfection amount of the corresponding plasmid to determine the relative expression levels.

#### Determination of the cAMP levels in cells

To examine the constitutive activity of PfSR10, we transfected HEK293 cells with different concentrations of the receptor, including PfSR10, ADGRG2, ADGRG3. For measuring the anti-malarial drugs, bradykinin or des-Arg9-bradykinin-induced inhibition of forskolin-stimulated cAMP level in HEK293 cells or COS-7 cells, the receptor encoding plasmid (PfSR10 or its mutants) or empty plasmid (pcDNA3.1) was co-transfected with the GloSensor plasmid and incubated in an incubator at 37°C with 5% CO2 for 24 h. The transfected cells were then inoculated on 96-well plates (at a density of 4 × 10^4^ / well) and incubated in incubators under the same conditions for another 24 hours. Cells were precultured with serum-free medium (Gibco) containing 5% DILUTED GloSensor™cAMP reagent Reserve solution (Progema) for 2 h. The base cAMP signal was directly measured using a multimode tablet reader (PerkinElmer Envision). At the same time, the cAMP signal induced by DHA, PIP-TT, PQ, CQ bradykinin or des-Arg9-bradykinin was detected after adding agonists into the corresponding Wells. Finally, the s-shaped dose response function in GraphPad Prism 7.0 was used to analyze the data.

For determination of the cAMP levels in the *Plasmodium falciparum*, *P. falciparum*-infected human erythrocyte s at trophozoite stage, synchronized with sorbitol as previously described.^47^ The *P*. *falciparum*-infected cells were subject to the 10 μM DHA treatment or not for 1 h after synchronization in the presence of 3 μM of Fsk. *P. falciparum* parasites were isolated at the trophozoite stage.^48^ *P*. *falciparum* parasites were washed twice with PBS and resuspended at a concentration of 5×10^6^ cells/ml^-1^ in the same buffer and incubated for 15 min with 10 μM DHA in the presence 300 nM IBMX to prevent cAMP degradation. After treatment, *P. falciparum* parasites were disrupted repeatedly by freeze-thaw, sedimented by centrifugation at 600 g for 10 min at 4°C and the supernatant was collected and subjected to cAMP measurement using cAMP ELISA Kit (R&D systems) according to manufacturer’s instructions.

#### ELISA assay to determine the IP3 level

To measure the IP3 levels in the *Plasmodium falciparum*. *P. falciparum* parasites were collected as described above. In brief, after 1 h-treatment 1h with 10 μM of DHA, *P. falciparum* parasites were isolated at the trophozoite stage and washed twice with PBS. Then P. falciparum parasites were disrupted repeatedly by freeze-thaw, sedimented by centrifugation at 600 g for 10 min at 4°C. The supernatant was collected and IP3 levels were determined using IP3 ELISA Kit (Hengyuan Biotechnology, Shanghai) according to manufacturer’s instructions.

#### FlAsH BRET assay

To characterize the binding properties of different ligands (CQ, DHA, PIP-TT, PQ, bradykinin or des-Arg9-bradykinin) to PfSR10, a FlAsH BRET assay was performed as previously described.^29^^,^^32^^,^^49^ We seeded HEK293 cells or COS-7 cells in 6-well plate were transfected with PfSR10 FlAsH BRET sensors (S1 to S6 as indicated in Figure 4B) and incubated for 48 h at 37°C in 5% CO2. The overexpressed PfSR10 sensors cells were washed with FlAsH BRET buffer (25 mM HEPES, 1 mM CaCl2, 140 mM NaCl, 2.7 mM KCl, 0.9 mM MgCl2, 0.37 mM NaH2PO4, 5.5 mM D-glucose, 12 mM NaHCO3) and incubated with 2.5 μM FlLAsH EDT2 (pre-diluted in FLAsH BRET buffer) for 40min at 37°C in 5% CO2. The cells were washed twice, resuspended with FlAsH BRET buffer, and seeded into a 96-well plate at a density of 5x10^4^ per well. After adding increasing concentrations of ligand (CQ, DHA, PIP-TT, PQ, bradykinin or des-Arg9-bradykinin) together with luciferase substrate coelenterazine-h (5 μM), the BRET signals between Nluc (440-480 nm) and FlAsH fluorescent moiety (525-585 nm) emissions were examined by a Mithras LB 940 Multimode Microplate Reader.

#### BRET assay for G protein activation measurement

To characterize the G protein signalling properties of PfSR10, G protein BRET probes were generated according to previous publication.^50^ The Gqo BRET probes were generated by replacing the six amino acids of the C- terminus of Gq -RlucII with those from G_oA1_, creating a chimeric Gqo-RlucII subunit.^51^ HEK293 cells or COS-7 cells were co-transfected with PfSR10 and various G protein probes (Gi, Gq and Gqo). Forty-eight hours post-transfection, cells were washed twice with HBSS, resuspended with HBSS buffer, and seeded into a 96-well plate at a density of 5x10^4^ per well. After adding increasing concentrations of ligand (CQ, DHA, PIP-TT, PQ, Bradykinin, des-Arg9-bradykinin, bradykinin-Arg-FITC, FITC-bradykinin) together with luciferase substrate coelenterazine 400a (5 μM, Cayman), the BRET signals between Nluc (440-480 nm) and GFP2 (510 nm) emissions were examined by a Mithras LB 940 Multimode Microplate Reader (Berthold Technologies). The BRET signal was calculated as the ratio of the GFP2 emission to Nluc emission.

#### Immunoprecipitation

Sf9 cells were infected with baculovirus encoding Flag-PfSR10-Δ375AA (N-terminal truncation of 375 amino acids). The purification and acquisition of the protein were described above. After purification, Anti-flag beads are incubated with M1 and placed at 4°C for 2 hours to generate Flag-PfSR10 beads. Human serum and erythrocyte were obtained from a healthy anonymous human donor. Blood was taken to heparin for anticoagulant management and separated the serum and red blood cells. The red blood cells were centrifuged at 500 g for 5 min at room temperature, then washed two times by 150 mM NaCl solution. The isolated red blood cells were completely lysed with 1%NP40 cell lysis buffer. Subsequently, erythrocyte lysates and serumwere incubated with Flag-PfSR10 coupling beads or M1 anti-Flag empty beads at 4°C for 2 h, respectively. The beads were washed with HEPES buffer containing 0.05% (W/V) LMNG, 0.1% (W/V) CHS, 2.5 mg/mL leukin and 2.5 mg/mL benzamidine. The PfSR10 or M1 beads-binding proteins were eluted with 20 mM HEPES, 100 mM NaCl, 0.05% LMNG, 0.1% CHS, 5 mM EGTA and 0.2 mg/mL FLAG peptide buffer. The eluted proteins of PfSR10 or M1 beads group were decomposed by SDS-PAGE and then silver stained using a Fast Silver staining kit (Beyotime, Shanghai, China) according to the manufacturer’s instructions. The PfSR10 or M1 beads group samples were digested overnight with trypsin. The peptides were desalted and dried in a rapid vacuum. The PfSR10 or M1 beads group samples were suspended in 10 μL buffer A (0.1% FA), separated by HIGH performance liquid chromatography (Easy nLC 1000-Thermo Scientific) and then injected into mass spectrometry (LTQ Orbitrap Elite-Thermo Scientific). The typical conditions of mass spectrometry are 2 kV spray voltage and 275°C capillary temperature. A full scanning mass spectrum with a resolution of 240,000 has been obtained in an orbital well (M /z 300∼1600). The obtained MS data files were processed using Proteome Discoverer 1.4 (Thermo Scientific) software and retrieved into the UniProt Pig (Sus Scrofa) Proteome database.

For further investigate the formation of the PfSR10-Gq complex, Sf9 cell pellets infected with PfSR10 and Gq heterotrimer were treated with procedures similar to that for PfSR10-Gi complex. Immuno-staining against PfSR10, Gq, Gβ and Gγ were performed using anti-FLAG (Sigma, F1804), anti-Gq (Proteintech, Catalog#27264-1-AP), anti-Gβ (Proteintech, 10247-2-AP), and anti-Gγ (Proteintech, Cat No#11693-1-AP) specific antibodies with 1:1000 dilution, respectively. The HRP-conjugated goat anti-rabbit IgG (Sigma Aldrich, Catalog#A6154) or HRP-conjugated goat anti-mouse IgG (Sigma Aldrich, Catalog#A0216) were used as the secondary antibody at a 1:2500 dilution.

### Quantification and statistical analysis

All quantitative data are presented in the figures as individual data points with the mean ± SEM (Standard Error of the Mean), unless otherwise specified in the figure legends. The exact value of n for each experiment, and what n represents (e.g., number of independent experiments, number of animals, or number of technical replicates), is provided in the corresponding figure legends. For the comparison between two groups, an unpaired two-tailed Student’s t-test was used. Differences between multiple groups with one independent variable were determined using one-way ANOVA. Based on the variance characteristics and experimental design of each dataset, this was followed by Tukey’s test, or Dunnett’s post hoc test for multiple comparisons, as specified in the figure legends. Statistical analysis was performed using GraphPad Prism 8 software. Significance was defined as ∗*P* < 0.05, ∗∗*P* < 0.01, ∗∗∗*P* < 0.001. No methods were used to determine whether the data met assumptions of the statistical approach. No power analysis was performed to determine the sample size.
