# Supplementary material for: Identification of antimalarial drugs and bradykinin as ligands of the Plasmodium membrane protein PfSR10
Source: iScience. 2025 Oct 21;28(11):113807. doi: 10.1016/j.isci.2025.113807 (PMC12639566; doi:10.1016/j.isci.2025.113807)
Supplement: Document S1. Figures S1–S9 and Tables S1–S9 [file mmc1.pdf]

iScience, Volume *28*

Supplemental information

Identification of antimalarial drugs and bradykinin as ligands of the *Plasmodium* membrane protein PfSR10

Dan Jiang, Xin Wen, Ji-Fei Han, Qiu-Xia Cheng, Ru Zhang, Yuan Zheng, Kai Zheng, Shao-Hui Huang, Jia-Yuan Chen, Su-Wen Li, Zhi-Shuai Yang, Bing Han, Lu Tie, Fan Yang, Peng-Ju Zhang, Peng Xiao, Hui Lin, Xiao Yu, and Jin-Peng Sun

Supplementary Information File for

Identification of anti-malaria drugs and bradykinin as ligands of PfSR10

This supplementary Information File includes:

Supplemental Figures 1-9

Supplemental Tables 1-9

Supplemental Figure 1. Constitutive activities of PfSR10.

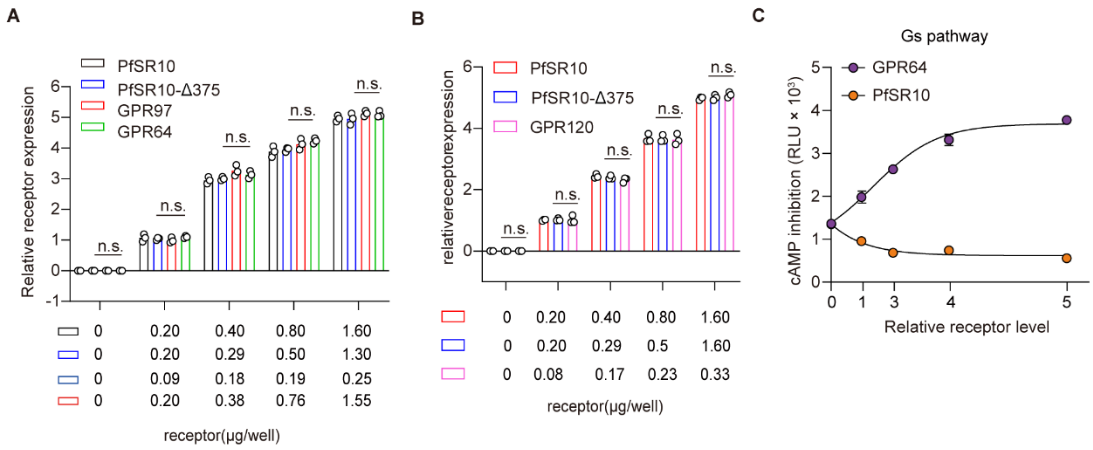

Supplemental Figure 1. Constitutive activities of PfSR10. Related to Figure 1.

A-B. ELISA experiments showing similar expression levels of PfSR10, PfSR10-Δ375, GPR64, GPR97, GPR120 when HEK293 cells were transiently transfected with indicated amounts of plasmids. Values are mean ± SEM from three independent experiments performed in triplicates. Comparison between PfSR10 and PfSR10-Δ375, GPR64, GPR97, GPR120 were determined by one-way ANOVA with Tukey's test. n.s., no statistical significance.

C. Glosensor-cAMP assay showing the constitutive activity of PfSR10 and GPR64. The individual receptors coupling to Gs were examined at similar receptor expression levels as indicated in Supplemental Figure1A. Data from three independent experiments performed in triplicates.

Supplemental Figure 2. Chemical structures of the potential antimalarial compounds investigated in this study.

A

Ligand	Abbreviations	Chemical structure
Quinine	QN	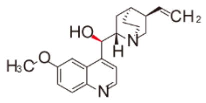
Quinidine	QND	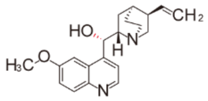
Euquinine	END	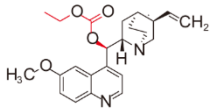
Mefloquine hydrochloride	MQ	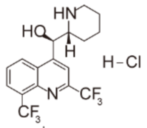
Primaquine diphosphate	PQ	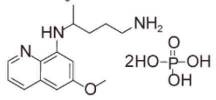
Chloroquine	CQ	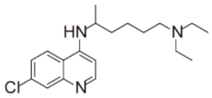
Piperaquine	PIP	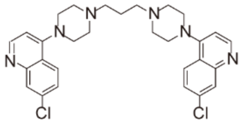
Piperaquine tetraphospha tetetrahydrate	PIP-TT	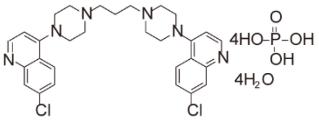

Ligand	Abbreviations	Chemical structure
Artemisinin	ART	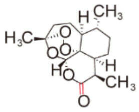
Dihydroartemisinin	DHA	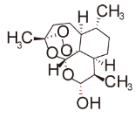
Artemether	ARTM	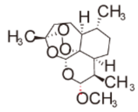
Artesunate	ATS	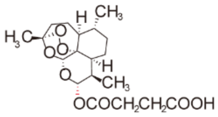
Atovaquone	ATO	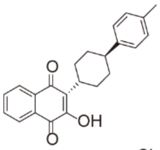
Pyrimethamine	PYM	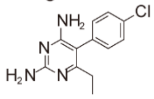
Lumefantrine	LUM	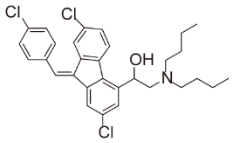

Supplemental Figure 2. Chemical structures of the potential antimalarial compounds investigated in this study. Related to Figure 2.
A. Two-dimensional representation of chemical structures of potential antimalarial drugs used in this study.

Supplemental Figure 3. Screening anti-malaria drugs ligands of PfSR10.

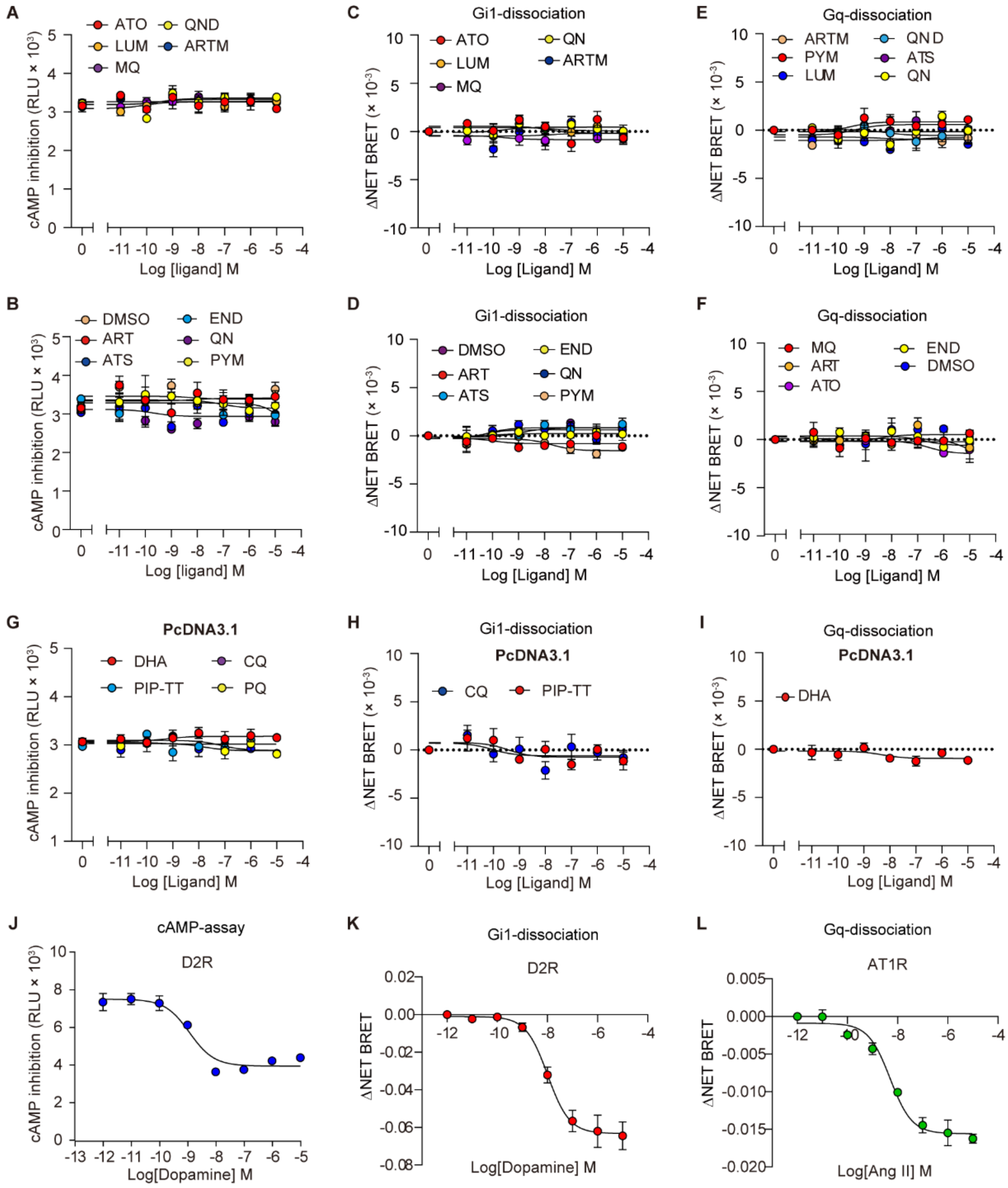

Supplemental Figure 3. Screening anti-malaria drugs ligands of PfSR10. Related to Figure 2.

A-B. Representative dose-response curve of the anti-malaria drugs induced cAMP inhibition in HEK293 cells overexpressing wild type PfSR10 using Glosensor assay. Data from three independent experiments performed in triplicates (n=3).

C-F. Representative dose-response curve of the anti-malaria drugs induced Gi1 (C, D) or Gq (E, F) dissociation signal in HEK293 cells overexpressing wild type PfSR10. Data from three independent experiments performed in triplicates(n=3).

G-I. Representative dose-response curve of four anti-malaria drugs induced Gi2 (G), Gi3 (H) or Gqo (I) dissociation signal in HEK293 cells overexpressing wild type PfSR10. Data from three independent experiments performed in triplicates(n=3).

J-L. Representative concentration-dependent curves of CQ-, DHA-, PIP-TT-, and PQ-induce cAMP inhibition (J), CQ- and PIP-TT -induce Gi1 (K) or DHA-induce Gq (L) dissociation in empty vector (pcDNA3.1)-overexpressing HEK293 cells. Data were from three independent experiments(n=3).

M-O. Representative concentration-dependent curves of DA- and AngII- induce cAMP inhibition (M), Gi1 (N) or Gq (O) dissociation in D2R- or AT1R- overexpressing HEK293 cells. Data were from three independent experiments (n=3).

Supplemental Figure 4. Cryo-EM reconstructions of the PfSR10-Gi-scFv16 complex.

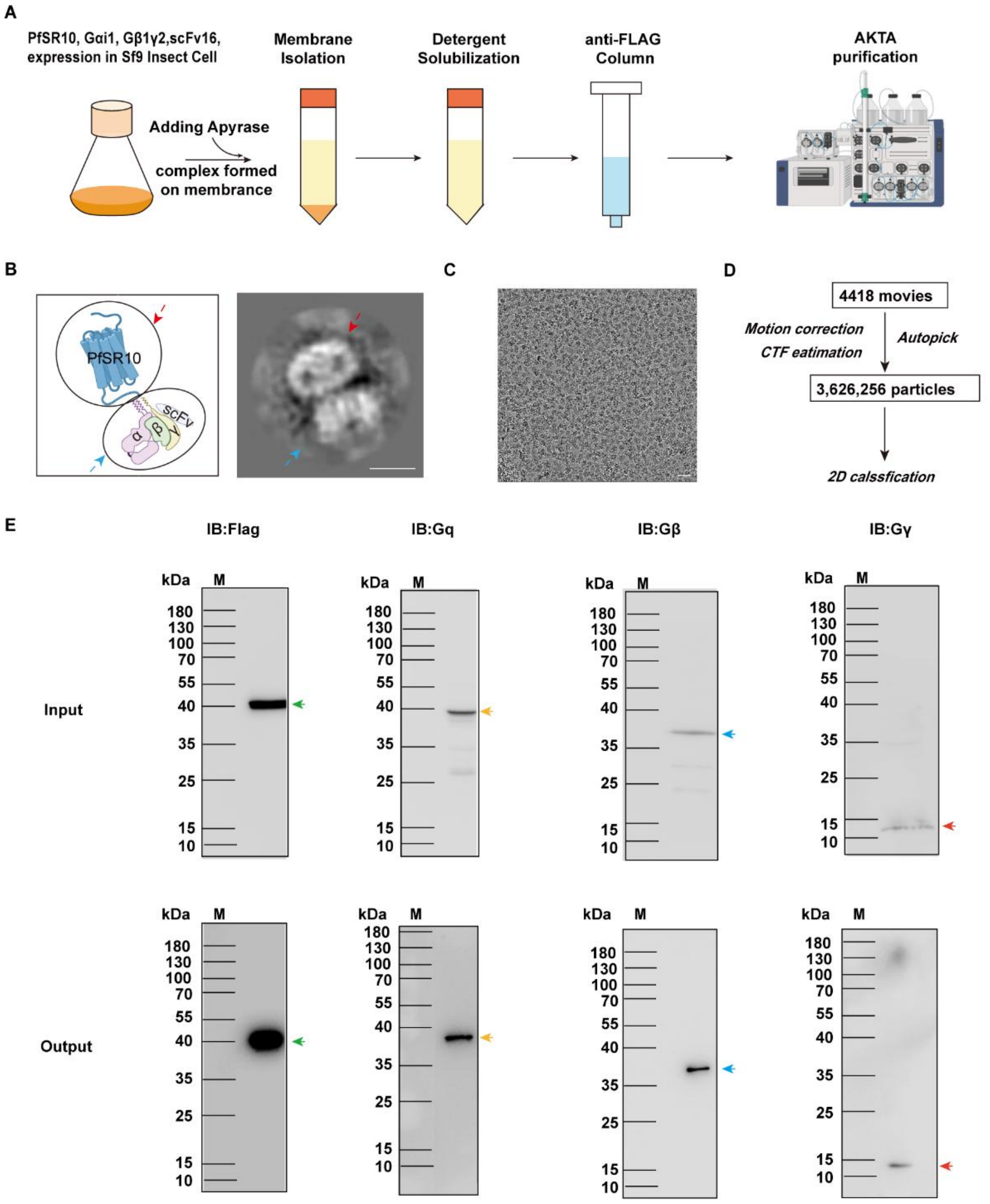

Supplemental Figure 4. Cryo-EM reconstructions of the PfSR10-Gi-scFv16 complex. Related to Figure 2.

A. Purification scheme for the PfSR10-Gi-scFv16 complex for cryo-EM analysis.

B. Schematic model containing the 2D model/crystal structures explaining what are each of particles and the general assembling of the complex. And representative 2D class averages of PfSR10-Gi-scFv16 complex clearly showed the coupling of Gi hetertrimer to PfSR10. Scale bar, 5 nm. PfSR10 is indicated by the red arrows, and the trimeric complex formed by Gαi1: Gβ1γ2: scFv16 is indicated by the blue arrows.

C. Representative cryo-EM micrograph (scale bar: 30 nm) of the PfSR10-Gi-scFv16 complex.

D. Schematic flow chart of the image processing steps for the PfSR10-Gi-scFv16 complex.

E. Western Blot analysis of PfSR10-Gq-scFv16 complex. Immunoblot demonstrating the presence of the FLAG-tagged component (epitope: DYKDDDDK), Gαq (G protein subunit alpha q, encoded by *GNAQ*), Gβ (G protein subunit beta, encoded by *GNB* gene family), and Gγ (G protein subunit gamma, encoded by *GNG* gene family) within the purified PfSR10-Gq-scFv16 complex. Molecular weight markers (kDa) are indicated on the left.

.

Supplemental Figure 5. The extracellular Conformational changes of PfSR10 within four anti-malaria drugs stimulation.

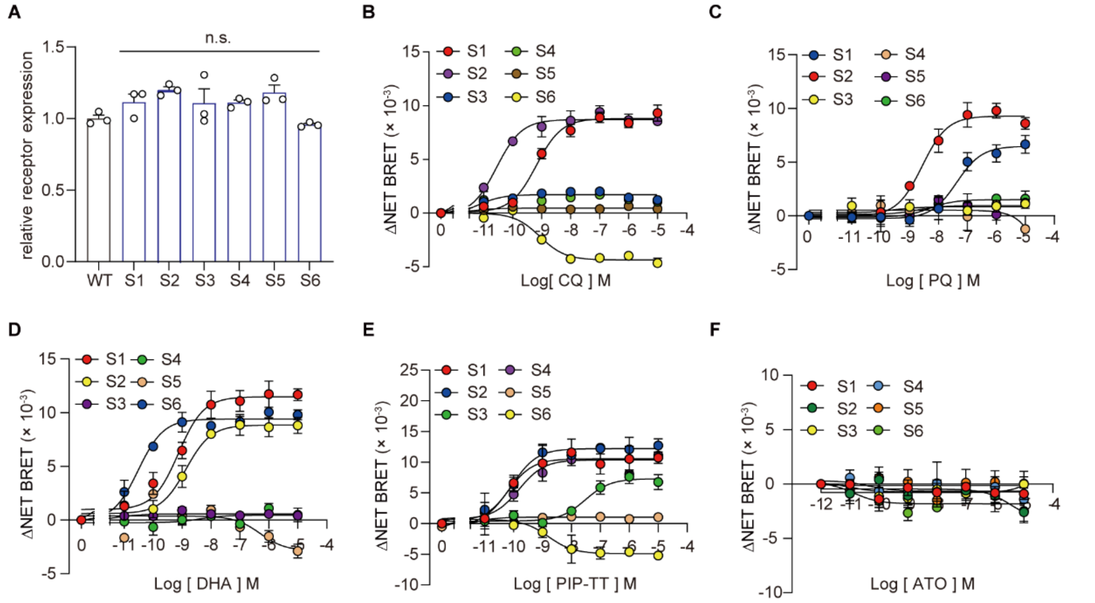

Supplemental Figure 5. The extracellular Conformational changes of PfSR10 within four anti-malaria drugs stimulation. Related to Figure 3.

A. ELISA experiments to determine the expression levels of the wild type PfSR10 and six FlAsH motif incorporated FlAsH-BRET sensors. Values are mean ± SEM from three independent experiments performed in triplicates. ns, no significance; Comparison between PfSR10-WT-Nluc and its mutant. All data were analysed by two-sided one-way ANOVA with Turkey test.

B-F. Representative dose-response curves of six PfSR10 FlAsH-BRET sensors in response to, CQ (B), PQ (C), DHA (D), PIP-TT (E) and ATO (F) stimulation. Data from three independent experiments performed in triplicates (n=3).

Supplemental Figure 6. Binding model of DHA with PfSR10.

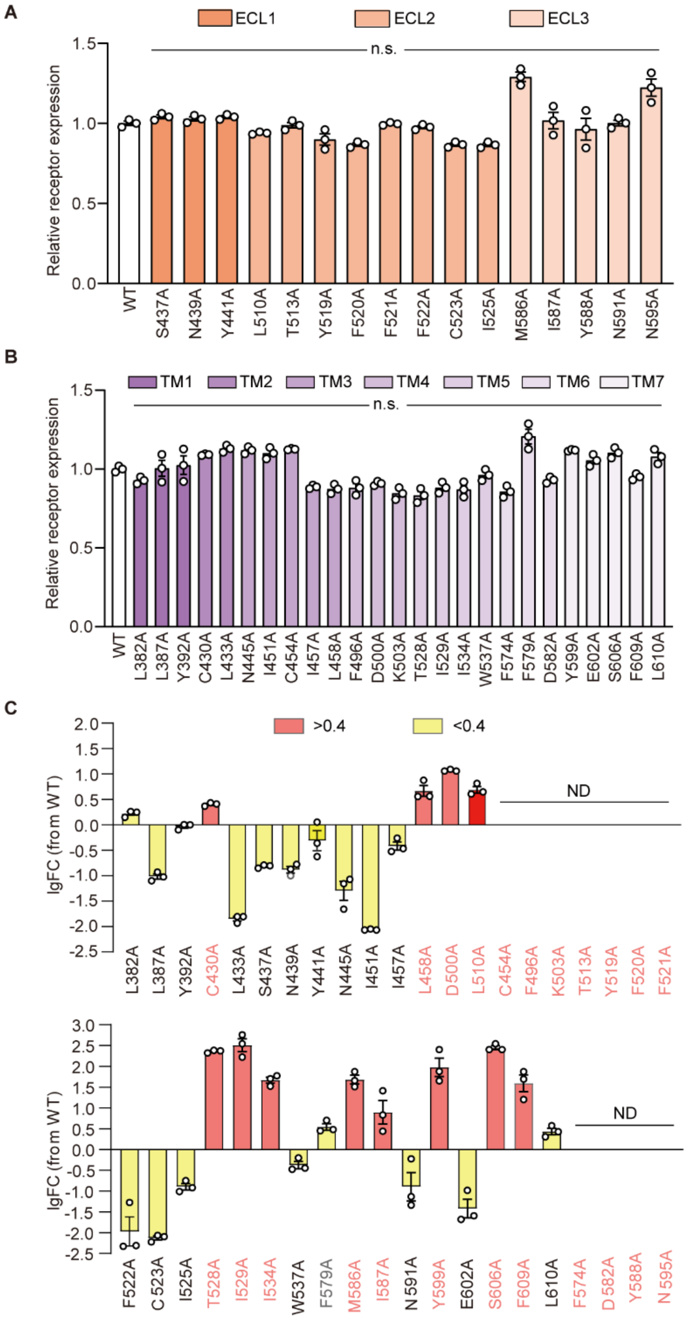

Supplemental Figure 6. Binding model of DHA with PfSR10. Related to Figure 4.
A-B. Relative expression levels of wild type PfSR10 and alanine mutants of putative binding sites at seven transmembrane domains and three extracellular loops were measured by ELISA assay. Data are from three independent experiments (n=3).
C. The coupling efficiency for DHA-induced cAMP inhibition of PfSR10 and alanine mutants. Bars represent differences in calculated EC50 values for each mutant relative to the WT receptor. A fold change of 2.5 is adopted as the cut-off for charactering the efficiency deterioration. Data are from three independent experiments (n=3).

Supplemental Figure 7. Activation of PfSR10 from different species by anti-malaria drugs.

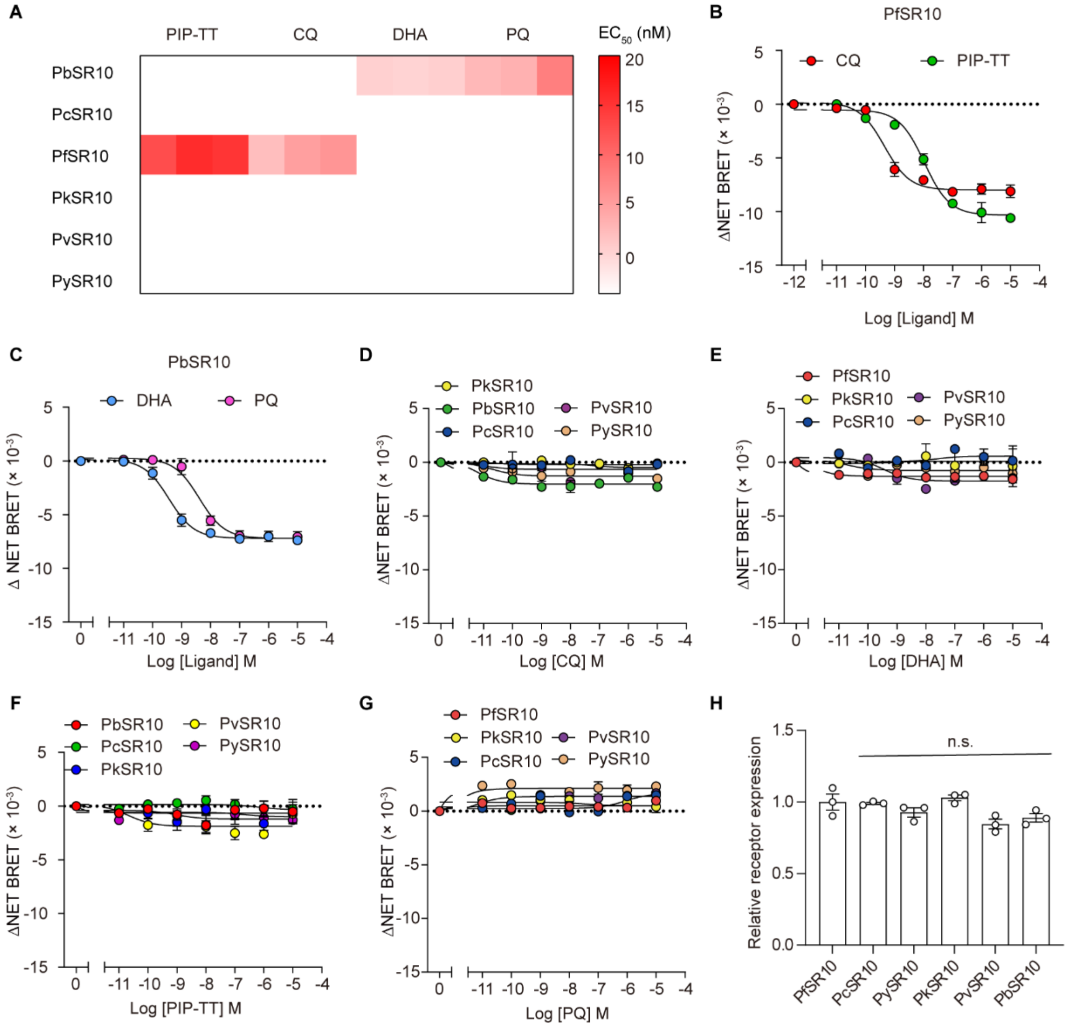

Supplemental Figure 7. Activation of PfSR10 from different species by anti-malaria drugs. Related to Figure 5.

A. Heatmap representing the Gi dissociation induced by anti-malaria drugs in HEK293 cells overexpressing SR10 from different species. The EC50 values were calculated to characterize the coupling efficiency between six different species SR10 and anti-malaria drug. The heatmap are generated base on the data shown Supplemental Figure 6B-C. Data were from three independent experiments (n=3).

B-C. Representative concentration-dependent curves of anti-malaria drug-stimulated Gi dissociation in HEK293 cells with overexpressing different species SR10. Data were from three independent experiments (n=3).

D-G. Representative concentration-dependent curves of anti-malaria drugs-stimulated Gi dissociation in HEK293 cells with overexpressing six different species SR10. Data were from three independent experiments (n=3).

H. ELISA experiments to determine the expression levels of PfSR10 and other five different species SR10. Values are mean ± SEM from three independent experiments performed in triplicates. ns, no significance; Comparison between PfSR10 and other five different species SR10. All data were analysed by two-sided one-way ANOVA with Turkey test.

Supplemental Figure 8. Coupling of PfSR10 to G protein in response to Bradykinin and Des-Arg9-Bradykinin.

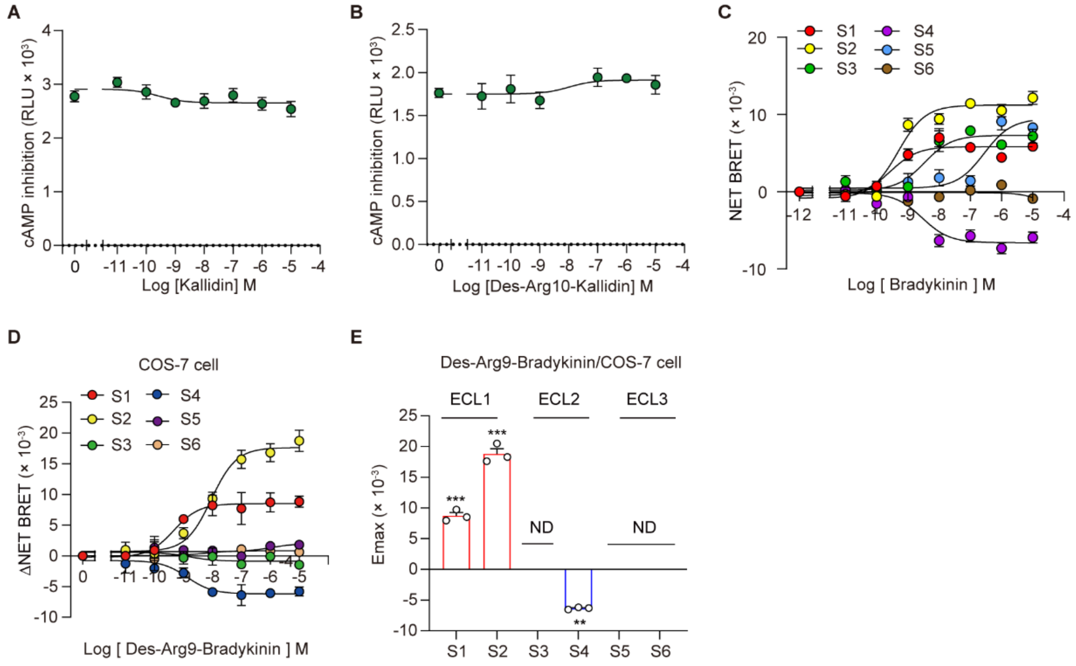

Supplemental Figure 8. Coupling of PfSR10 to G protein in response to Bradykinin and Des-Arg9-Bradykinin. Related to Figure 6.

A-B. Representative dose-response curve of Kallidin (A) or Des-Arg10-Kallidin (B) induced cAMP inhibition in HEK293 cells overexpressing wild type PfSR10 using Glosensor assay. Data from three independent experiments performed in triplicates (n=3).

C-F. Representative dose-response curve of Bradykinin or Des-Arg9-Bradykinin induced Gi2 (C), Gi3 (D) or Gqo (E) dissociation signal and Des-Arg9-Bradykinin stimulated cAMP inhibition (f) in HEK293 cells overexpressing wild type PfSR10. Data from three independent experiments performed in triplicates (n=3).

G-H. Representative dose-response curves of six PfSR10 FlAsH-BRET sensors in response to Bradykinin (G) or Des-Arg9-Bradykinin (H) stimulation. Data from three independent experiments performed in triplicates (n=3).

Supplemental Figure 9. Molecular basis of Bradykinin recognition and species-specific activation of SR10.

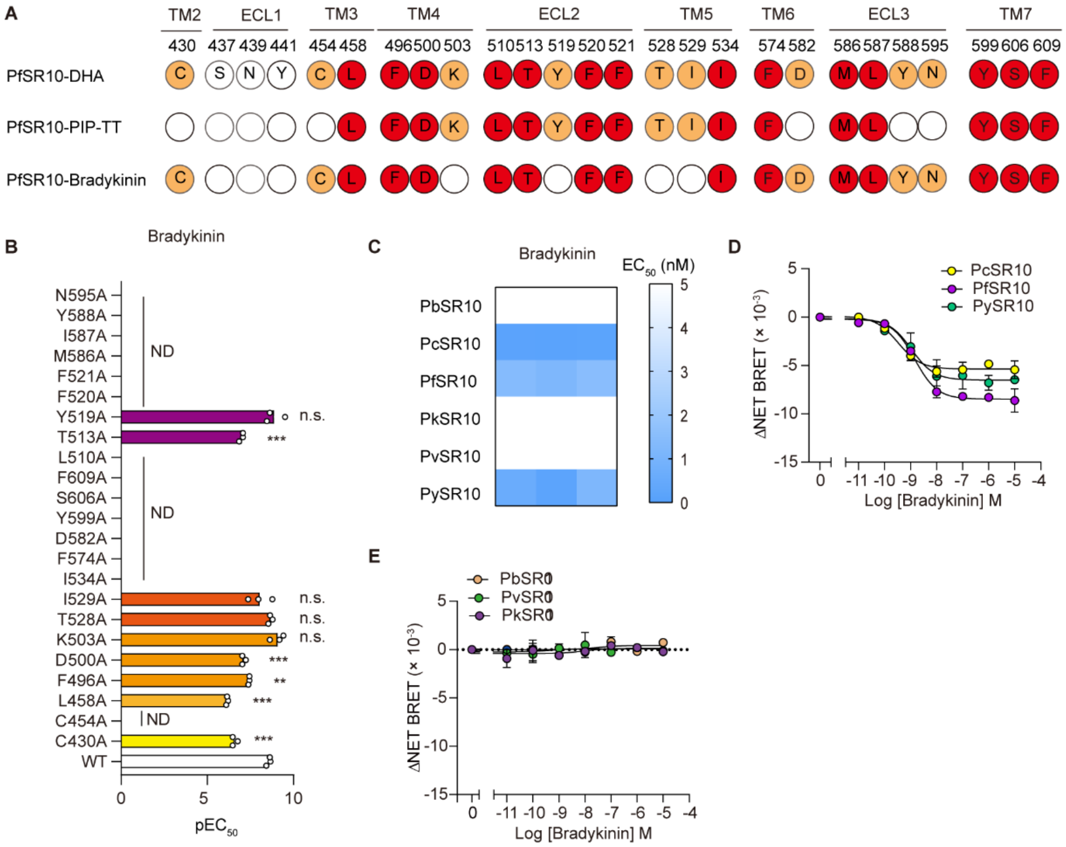

Supplemental Figure 9. Molecular basis of Bradykinin recognition and species-specific activation of SR10. Related to Figure 6.

A. The residues in PfSR10 that contact with DHA, PIP-TT or Bradykinin. Residues of which the mutation led to decreased potency for three ligands- (inducing cAMP inhibition) were colored in red, while residues of which the mutation led to decreased potency for any two ligand-stimulated cAMP inhibition were colored in orange.

B. Bar graph showing the pEC50 of Bradykinin in the activation of wild-type (WT) or mutant PfSR10. Each value is the mean ± SEM of three independent experiments (n = 3). The bars indicate mean ± SEM values. All data were statistically analyzed using one-way ANOVA with Dunnett's post hoc test, with significance levels indicated as *P < 0.05, **P < 0.01, **P < 0.001.

C. Heatmap representing the Gi dissociation induced by Bradykinin in HEK293 cells overexpressing SR10 from different species. The EC50 values were calculated to characterize the coupling efficiency between six different species SR10 and Bradykinin. The heatmap are generated base on the data shown Supplemental Figure 8D. Data were from three independent experiments (n=3).

D-E. Representative concentration-dependent curves of Bradykinin-stimulated Gi dissociation in HEK293 cells with overexpressing different species SR10. Data were from three independent experiments (n=3).

.

Supplementary Table 1. Cryo-EM data collection of PfSR10-Gi complex. Related to Figure 2 and Supplemental Figure 3.

Parameters	PIP-TT-PfSR10-Gi complex
**Data collection and processing**	
EM equipment	FEI Titan krios
Voltage (kV)	300
Detector	Gatan K2
Electron exposure (e–/Å^2)	60(32 frames)
Defocus range (μm)	-1 to -2
Pixel size (Å)	1.08
Particles	3,626,256

Supplementary Table 2. PfSR10-interacting partners identified by LC-MS/MS analysis and classified as secretory proteins in serum. Related to Figure 6.

GENE SYMBOL	PROTEIN IDENTITY	FOLD INTENSITY PfSR10/CONTROL	NUMBER OF UNIQUE PEPTIDES	COVERAGE (%)
**Proteins present in PfSR10 group only**				
C4B	C4a anaphylatoxin	N/A	45	37.81
FGG	Fibrinogen gamma chain	N/A	15	38.65
CFB	Complement factor B	N/A	8	10.99
PKM	Pyruvate kinase (Fragment)	N/A	8	18.35
IGHV3OR16-9	Immunoglobulin heavy variable 3/OR16-9 (non-functional)	N/A	1	31.25
F2	Activation peptide fragment 1	N/A	4	8.92
IGKV3-15	Immunoglobulin kappa variable 3-15	N/A	1	26.09
TAGLN2	Transgelin-2 (Fragment)	N/A	3	21.93
KRT13	Keratin, type I cytoskeletal 13	N/A	1	6.99
EEF1A1	Elongation factor 1-alpha	N/A	3	7.04
APOC2	Apolipoprotein C-II	N/A	2	29.70
IGHV3OR16-12	Immunoglobulin heavy variable 3/OR16-12 (non-functional) (Fragment)	N/A	2	18.80
JCHAIN	Immunoglobulin J chain (Fragment)	N/A	3	38.57
IGKV2D-28	Immunoglobulin kappa variable 2D-28	N/A	1	19.61
PLTP	Phospholipid transfer protein	N/A	1	2.23
APOF	Apolipoprotein F	N/A	1	4.29
LGALS3BP	Galectin-3-binding protein	N/A	1	22.92
SERPINF2	Alpha-2-antiplasmin (Fragment)	N/A	1	5.10
COLEC11	Collectin-11	N/A	1	4.80
FCGBP	IgGFc-binding protein	N/A	1	0.55
RPS5	40S ribosomal protein S5	N/A	1	6.72
IGLV3-10	Immunoglobulin lambda variable 3-10	N/A	1	6.96
KLKB1	Plasma kallikrein	N/A	1	1.75
IGLV2-11	Immunoglobulin lambda variable 2-11	N/A	1	6.72
**Proteins presence in both groups but with an intensity ratio of at least 1.5-Fold increase**				
IGLV8-61	Immunoglobulin lambda variable 8-61 OS=Homo sapiens	1.998644986	2	14.75
ITIH3	Inter-alpha-trypsin inhibitor heavy chain H3 OS=Homo sapiens	2.100671141	2	3.13
CD5L	CD5 antigen-like OS=Homo sapiens	1.937093275	3	8.93
IGHV3-7	Immunoglobulin heavy variable 3-7 OS=Homo sapiens	1.900526624	3	32.48
APOD	Apolipoprotein D OS=Homo sapiens	1.645121951	5	26.98
C4BPB	C4b-binding protein beta chain OS=Homo sapiens	3.445378151	3	12.30
APOA5	Apolipoprotein A-V OS=Homo sapiens	1.776422764	2	4.37

Supplementary Table 3. PfSR10-interacting partners identified by LC-MS/MS analysis and classified as membrane proteins in serum. Related to Figure 6.

GENE SYMBOL	PROTEIN IDENTITY	FOLD INTENSITY PfSR10/CONTROL	NUMBER OF UNIQUE PEPTIDES	COVERAGE (%)
**Proteins present in PfSR10 group only**				
ACTN1	Alpha-actinin-1	N/A	15	22.51
MRGPRX2	Mas-related G-protein coupled receptor member X2	N/A	2	8.18
HTR4	5-hydroxytryptamine receptor 4	N/A	2	8.09
ITGB3	Integrin beta	N/A	3	8.13
CAP1	Adenylyl cyclase-associated protein 1 (Fragment)	N/A	2	12.64
AFG3L2	AFG3-like protein 2	N/A	2	2.89
PHB2	Prohibitin (Fragment)	N/A	2	8.92
ADGRL1	Adhesion G protein-coupled receptor L1	N/A	1	0.75
GPR65	Psychosine receptor	N/A	2	5.34
CFL1	Cofilin, non-muscle isoform (Fragment)	N/A	1	35.44
GALNT10	Polypeptide N-acetylgalactosaminyltransferase 10	N/A	1	3.96
IZUMO1R	Sperm-egg fusion protein Juno	N/A	1	4.80
PLXNB3	Plexin-B3	N/A	1	0.37
**Proteins presence in both groups but with an intensity ratio of at least 1.5-Fold increase**				
ATP5F1A	ATP synthase subunit alpha, mitochondrial OS=Homo sapiens	2.411347518	9	17.00
GPR4	G-protein coupled receptor 4 OS=Homo sapiens	4.005181347	1	7.73

Supplementary Table 4. PfSR10-interacting partners identified by LC-MS/MS analysis and classified as cytoplasmic proteins in serum. Related to Figure 6.

GENE SYMBOL	PROTEIN IDENTITY	FOLD INTENSITY PfSR10/CONTROL	NUMBER OF UNIQUE PEPTIDES	COVERAGE (%)
**Proteins present in PfSR10 group only**				
ATP5F1B	ATP synthase subunit beta (Fragment)	N/A	11	43.65
GSN	Actin-depolymerizing factor	N/A	12	18.18
TPM4	Tropomyosin alpha-4 chain	N/A	7	33.87
TPM1	Tropomyosin alpha-1 chain	N/A	2	15.51
H2AZ2	Histone H2A OS=Homo sapiens	N/A	2	13.11
SURF4	Surfeit locus protein 4	N/A	1	4.84
AMBP	Alpha-1-microglobulin (Fragment)	N/A	2	8.81
HADHA	Enoyl-CoA hydratase	N/A	1	1.24
ZYX	Zyxin (Fragment)	N/A	2	15.91
UBC	Polyubiquitin-C (Fragment)	N/A	2	24.16
TMED4	Transmembrane emp24 domain-containing protein 4	N/A	2	3.96
CALR	Calreticulin (Fragment)	N/A	1	6.07
NCOA6	Nuclear receptor coactivator 6	N/A	1	0.44
Figure4	Polyphosphoinositide phosphatase (Fragment)	N/A	1	2.28
PHB	Prohibitin (Fragment)	N/A	1	8.06
PRDX1	Peroxiredoxin-1	N/A	1	11.34
CANX	Calnexin	N/A	1	1.52
CCDC34	Coiled-coil domain-containing protein 34	N/A	1	2.41
SACM1L	Phosphatidylinositol-3-phosphatase SAC1 (Fragment)	N/A	1	4.28
FAM83H	Protein FAM83H (Fragment)	N/A	1	1.77
C8G	Complement component C8 gamma chain (Fragment)	N/A	1	12.31
HSP90B2P	Putative endoplasmin-like protein	N/A	1	2.51
PCCA	Propionyl-CoA carboxylase alpha chain, mitochondrial (Fragment)	N/A	1	5.76
TMED9	Transmembrane emp24 domain-containing protein 9	N/A	1	3.83
TECR	Very-long-chain enoyl-CoA reductase	N/A	1	2.92
ARPC4-TTLL3	ARPC4-TTLL3 readthrough	N/A	1	19.15
HYDIN	Hydrocephalus-inducing protein homolog	N/A	1	4.41

	(Fragment)			
OGFOD1	Prolyl 3-hydroxylase OGFOD1	N/A	1	1.40
STT3B	Dolichyl-diphosphooligosaccharide--protein glycosyltransferase subunit STT3B	N/A	1	0.97
UBE2Q2	Ubiquitin-conjugating enzyme E2 Q2 (Fragment)	N/A	1	8.79
ATP2A2	P-type Ca(2+) transporter (Fragment)	N/A	1	0.75
PDE3B	cGMP-inhibited 3',5'-cyclic phosphodiesterase B	N/A	1	3.06
MON2	Protein MON2 homolog	N/A	1	1.03
NAA15	N-alpha-acetyltransferase15,NatA auxiliary subunit	N/A	1	1.50
**Proteins presence in both groups but with an intensity ratio of at least 1.5-Fold increase**				
GNAQ	Guanine nucleotide-binding protein G(q) subunit alpha OS=Homo sapiens	2.53961136	4	16.99
GNAI1	Guanine nucleotide-binding protein G(i) subunit alpha-1 OS=Homo sapiens	3.858347386	9	45.76

Supplementary Table 5. PfSR10-interacting partners identified by LC-MS/MS analysis and classified as secreted proteins in erythrocyte. Related to Figure 6.

GENE SYMBOL	PROTEIN IDENTITY	FOLD INTENSITY PfSR10/CONTROL	NUMBER OF UNIQUE PEPTIDES	COVERAGE (%)
**Proteins present in PfSR10 group only**				
APOE	Apolipoprotein E	N/A	8	26.18
THBS1	Thrombospondin-1	N/A	8	8.03
CLU	Clusterin	N/A	7	20.49
YWHAH	14-3-3 protein eta	N/A	3	27.64
PSMD3	26S proteasome non-ATPase regulatory subunit 3	N/A	6	12.17
PPP2R1B	Serine/threonine-protein phosphatase 2A 65 kDa regulatory subunit A beta isoform	N/A	6	16.14
ATP6V1H	V-type proton ATPase subunit H	N/A	6	14.49
PON1	Serum paraoxonase/arylesterase 1	N/A	2	13.52
CALML5	Calmodulin-like protein 5	N/A	3	23.97
PLEK	Pleckstrin	N/A	2	8.29
C4B_2	C4a anaphylatoxin	N/A	3	1.94
S100A7	Protein S100-A7	N/A	2	21.78
RPLP2	60S acidic ribosomal protein P2	N/A	2	42.61
SERPINA3	Alpha-1-antichymotrypsin	N/A	2	7.09
IGKV3-15	Immunoglobulin kappa variable 3-15	N/A	1	7.83
SBSN	Suprabasin	N/A	1	9.15
C1QA	Complement C1q subcomponent subunit A	N/A	1	11.02
SERPINB4	Serpin B4 (Fragment)	N/A	1	5.69
HMGB2	High mobility group protein B2	N/A	1	5.26
RETN	Resistin	N/A	1	10.19
SERPINB1	Leukocyte elastase inhibitor	N/A	1	2.90
PSMD7	26S proteasome non-ATPase regulatory subunit 7	N/A	2	6.17
APOA4	Apolipoprotein A-IV	N/A	1	2.27
KNG1	Kininogen-1	N/A	1	1.40
MUC19	Mucin-19	N/A	1	0.23
**Proteins presence in both groups but with an intensity ratio of at least 1.5-Fold increase**				
APOB	Apolipoprotein B-100	19.00	57.00	14.81
GNL1	Guanine nucleotide-binding protein-like 1	2.00	12.00	23.72
PSMD2	26S proteasome non-ATPase regulatory subunit 2	2.00	12.00	17.84
EIF2S3	Eukaryotic translation initiation factor 2 subunit 3	2.50	10.00	22.03
PSMD1	26S proteasome non-ATPase regulatory subunit 1	2.00	8.00	10.81

CNP	2',3'-cyclic-nucleotide 3'-phosphodiesterase	1.75	7.00	16.63
GSN	Actin-depolymerizing factor	1.75	7.00	9.36
BPI	Bactericidal permeability-increasing protein	2.00	4.00	12.22
PF4	Platelet factor 4	2.00	4.00	42.57
BID	BH3-interacting domain death agonist	3.00	3.00	20.00
CSNK2B	Casein kinase II subunit beta	3.00	3.00	14.42
PSMD12	26S proteasome non-ATPase regulatory subunit 12	3.00	3.00	7.02
S100A12	Protein S100-A12	2.00	2.00	29.35
LGALS3	Galectin-3	3.00	3.00	14.40
PSMD14	26S proteasome non-ATPase regulatory subunit 14	2.00	2.00	6.45

Supplementary Table 6. PfSR10-interacting partners identified by LC-MS/MS analysis and classified as membrane proteins in erythrocyte. Related to Figure 6.

GENE SYMBOL	PROTEIN IDENTITY	FOLD INTENSITY PfSR10/CONTROL	NUMBER OF UNIQUE PEPTIDES	COVERAGE (%)
**Proteins present in PfSR10 group only**				
S100A6	Protein S100-A6	N/A	7	61.11
FLOT1	Flotillin-1	N/A	12	32.32
ABCC4	Multidrug resistance-associated protein 4	N/A	16	14.11
KEL	Kell blood group glycoprotein	N/A	13	24.18
FLNA	Filamin-A	N/A	10	6.35
ATP2B1	Plasma membrane calcium-transporting ATPase 1	N/A	1	11.97
PIEZO1	Piezo-type mechanosensitive ion channel component 1 OS=Homo sapiens	N/A	9	5.39
SLC29A1	Equilibrative nucleoside transporter 1 OS=Homo sapiens	N/A	6	14.04
FLOT2	Flotillin OS=Homo sapiens	N/A	8	17.52
ITGA2B	Integrin alpha-IIb	N/A	7	7.89
EEF1A1	Elongation factor 1-alpha 1	N/A	6	15.80
ATP5F1A	ATP synthase subunit alpha, mitochondrial	N/A	7	15.01
RAP1B	Ras-related protein Rap-1b	N/A	1	26.63
RHD	Blood group Rh(D) polypeptide	N/A	2	11.27
RAB8A	Ras-related protein Rab-8A	N/A	2	21.26
CFL1	Cofilin, non-muscle isoform (Fragment)	N/A	3	45.90
SLC14A1	Urea transporter 1	N/A	5	11.31
EHD1	EH domain-containing protein 1 (Fragment)	N/A	3	14.44
SLC40A1	Solute carrier family 40 member 1	N/A	2	3.50
RHCE	Blood group Rh(CE) polypeptide	N/A	1	11.47
XK	Membrane transport protein XK	N/A	4	9.68
AP2M1	AP-2 complex subunit mu	N/A	3	8.99
SNX9	Sorting nexin-9	N/A	2	3.53
SLC25A31	ADP/ATP translocase 4	N/A	1	6.35
ARL6IP5	PRA1 family protein 3	N/A	3	13.30
TBC1D24	TBC1 domain family member 24	N/A	2	3.22
NRAS	GTPase NRas	N/A	1	13.23
BSG	Basigin (Fragment)	N/A	2	27.38
EIF2B1	Translation initiation factor eIF-2B subunit alpha	N/A	2	5.90
EPB41L2	Band 4.1-like protein 2	N/A	1	2.27
DBNL	Drebrin-like protein	N/A	1	3.06

	(Fragment)			
HVCN1	Hydrogen voltage-gated channel 1 (Fragment)	N/A	1	7.01
SVIP	Small VCP/p97-interacting protein	N/A	1	14.29
ARF6	ADP-ribosylation factor 6	N/A	1	6.29
SLC16A1	Monocarboxylate transporter 1 (Fragment)	N/A	1	4.05
HTR4	5-hydroxytryptamine receptor 4	N/A	1	2.96
CYBRD1	Cytochrome b reductase 1	N/A	1	4.20
PHB2	Prohibitin (Fragment)	N/A	1	3.45
AP2S1	AP complex subunit sigma	N/A	1	6.56
CELSR3	Cadherin EGF LAG seven-pass G-type receptor 3	N/A	1	0.94
PLXNB3	Plexin-B3	N/A	1	0.52
TTLL5	Tubulin polyglutamylase TTLL5	N/A	1	0.62
**Proteins presence in both groups but with an intensity ratio of at least 1.5-Fold increase**				
ATP2B4	Plasma membrane calcium-transporting ATPase 4	10.50	11.00	21.84
OSBP2	Oxysterol-binding protein	2.00	2.00	2.52

Supplementary Table 7. PfSR10-interacting partners identified by LC-MS/MS analysis and classified as cytoplasmic proteins in erythrocyte. Related to Figure 6.

GENE SYMBOL	PROTEIN IDENTITY	FOLD INTENSITY PfSR10/CONTROL	NUMBER OF UNIQUE PEPTIDES	COVERAGE (%)
**Proteins present in PfSR10 group only**				
TUBB	Tubulin beta chain	N/A	2	53.6
TUBB2A	Tubulin beta-2A chain	N/A	1	48.54
EPB41	Band 4.1	N/A	1	33.76
TUBA4A	Tubulin alpha-4A chain	N/A	2	46.21
TUBA3C	Tubulin alpha-3C chain	N/A	2	46
TUBB1	Tubulin beta-1 chain	N/A	11	45.23
CSE1L	Exportin-2	N/A	16	19.05
ATP5F1B	ATP synthase subunit beta, mitochondrial	N/A	11	27.41
TPM1	Tropomyosin 1 (Alpha), isoform CRA_m	N/A	8	41.94
EIF4A1	Eukaryotic initiation factor 4A-I	N/A	9	26.85
KRT16	Keratin, type I cytoskeletal 16	N/A	2	19.24
KRT6B	Keratin, type II cytoskeletal 6B	N/A	1	17.38
CCT3	T-complex protein 1 subunit gamma	N/A	10	27.16
TCP1	T-complex protein 1 subunit alpha	N/A	8	16.19
COPS3	COP9 signalosome complex subunit 3	N/A	6	20.09
VAC14	Protein VAC14 homolog	N/A	8	13.17
METTL7A	Methyltransferase-like protein 7A (Fragment)	N/A	4	30
USP24	Ubiquitin carboxyl-terminal hydrolase 24	N/A	7	3.47
ARHGDIB	Rho GDP-dissociation inhibitor 2	N/A	7	45.77
HSPA5	Endoplasmic reticulum chaperone BiP	N/A	4	11.01
PSMB3	Proteasome chain 13 (Fragment)	N/A	6	55.17
HSD17B12	Very-long-chain 3-oxoacyl-CoA reductase	N/A	6	15.06
SACM1L	Phosphatidylinositol-3-phosphatase SAC1	N/A	6	11.78
HSPB1	Heat shock protein beta-1	N/A	5	37.56
MNDA	Myeloid cell nuclear differentiation antigen	N/A	5	15.23
TPM4	Tropomyosin alpha-4 chain	N/A	1	20.77
ALDOA	Fructose-bisphosphate aldolase (Fragment)	N/A	4	19.42
TNPO1	Transportin-1	N/A	3	6.46
PSMD11	26S proteasome non-ATPase regulatory subunit 11	N/A	3	9.48
EIF4B	Eukaryotic translation initiation factor 4B	N/A	2	3.93
TNPO3	Transportin-3	N/A	5	7.47

RILP	Rab-interacting lysosomal protein	N/A	3	11.47
RTN4	Reticulon	N/A	3	10.14
PRPSAP1	Phosphoribosyl pyrophosphate synthase-associated protein 1	N/A	3	9.55
ATG4A	Cysteine protease ATG4A	N/A	3	7.79
CPT1A	Carnitine O-palmitoyltransferase 1, liver isoform	N/A	3	4.14
WDR44	WD repeat-containing protein 44 (Fragment)	N/A	3	4.09
PSMD8	26S proteasome non-ATPase regulatory subunit 8	N/A	4	19.16
COPS7B	COP9 signalosome complex subunit 7b	N/A	3	16.09
SMC4	Structural maintenance of chromosomes protein	N/A	3	2.61
CSNK1A1	Casein kinase I isoform alpha	N/A	3	8.61
PURA	Transcriptional activator protein Pur-alpha	N/A	3	8.07
ATP2A3	Sarcoplasmic/endoplasmic reticulum calcium ATPase 3	N/A	3	3
WDR91	WD repeat-containing protein 91	N/A	1	1.69
CLTA	Clathrin light chain A	N/A	3	10.08
GLMN	Glomulin	N/A	2	4.71
CUL1	Cullin-1	N/A	3	3.54
SLC43A3	Solute carrier family 43 member 3 (Fragment)	N/A	2	4.83
SCAMP2	Secretory carrier-associated membrane protein 2	N/A	2	7.6
UBE4A	Ubiquitin conjugation factor E4 A	N/A	1	2.6
SLC25A5	ADP/ATP translocase 2	N/A	2	9.06
PDCD10	Programmed cell death protein 10 (Fragment)	N/A	2	9.9
PTGES3	Prostaglandin E synthase 3	N/A	2	18.13
MAP2K3	Dual specificity mitogen-activated protein kinase kinase 3	N/A	2	7.2
RDH11	Retinol dehydrogenase 11	N/A	2	8.49
PYCR3	Pyrroline-5-carboxylate reductase 3	N/A	2	8.03
USO1	General vesicular transport factor p115	N/A	2	2.29
TNPO2	Transportin-2	N/A	1	3.26
DCAF11	DDB1- and CUL4-associated factor 11	N/A	1	2.01
TLCD4	TLC domain-containing protein 4	N/A	4	14.07
CDK2	Cyclin-dependent kinase 2	N/A	2	10.5
HECTD3	E3 ubiquitin-protein ligase HECTD3	N/A	2	3.83

ARMC8	Armadillo repeat-containing protein 8 (Fragment)	N/A	2	5.97
SURF4	Surfeit locus protein 4	N/A	2	10.22
TMCC2	Transmembrane and coiled-coil domains protein 2	N/A	2	5.45
KRT80	Keratin, type II cytoskeletal 80	N/A	1	5.09
APRT	Adenine phosphoribosyltransferase (Fragment)	N/A	2	10.49
PPP2R5D	Serine/threonine-protein phosphatase 2A 56 kDa regulatory subunit	N/A	2	4.21
GYPA	Glycophorin-A	N/A	1	43.06
RHAG	Ammonium transporter Rh type A	N/A	2	6.36
ITGB3	Integrin beta	N/A	2	5.87
ATL3	Atlastin-3	N/A	2	4.21
DCTN1	Dynactin subunit 1	N/A	2	2.25
IPO11	Importin-11	N/A	2	2.09
ARIH2	E3 ubiquitin-protein ligase ARIH2	N/A	1	3.45
NP	N-acetylneuraminate lyase	N/A	2	5.63
MCTS1	Malignant T-cell-amplified sequence 1	N/A	1	11.05
SCFD1	Sec1 family domain-containing protein 1	N/A	3	5.92
MAP2K1	Dual specificity mitogen-activated protein kinase kinase 1	N/A	2	5.34
WIPI2	WD repeat domain phosphoinositide-interacting protein 2	N/A	1	5.07
IPO13	Importin-13	N/A	1	1.45
DCAF6	DDB1- and CUL4-associated factor 6	N/A	2	3.18
ATP2A2	P-type Ca(2+) transporter (Fragment)	N/A	2	1.82
AKAP7	A-kinase anchor protein 7 isoform gamma	N/A	1	3.45
CIAO2A	Cytosolic iron-sulfur assembly component 2A	N/A	1	6.88
RBX1	E3 ubiquitin-protein ligase RBX1	N/A	1	7.41
MT-ATP6	ATP synthase subunit a	N/A	1	4.42
ACKR1	Atypical chemokine receptor 1	N/A	1	6.55
SEC22B	Vesicle-trafficking protein SEC22b	N/A	1	4.65
TMEM245	Transmembrane protein 245	N/A	1	1.48
RAB21	Ras-related protein Rab-21	N/A	1	4.44
TMEM109	Transmembrane protein 109	N/A	1	4.94
YIPF6	Protein YIPF6	N/A	1	5.08

SLC25A3	Phosphate carrier protein, mitochondrial	N/A	1	3.7
VDAC3	Voltage-dependent anion-selective channel protein 3	N/A	1	3.89
RUVBL2	RuvB-like helicase	N/A	1	5.02
APOL3	Apolipoprotein L3	N/A	1	2.74
GYPA	Glycophorin-A (Fragment)	N/A	1	14.42
EEF1D	Elongation factor 1-delta	N/A	1	18.46
PGRMC2	Membrane-associated progesterone receptor component 2	N/A	1	6.28
TMCO1	Calcium load-activated calcium channel	N/A	1	10.89
TBCB	Tubulin-folding cofactor B (Fragment)	N/A	1	5.92
UNC13D	Protein unc-13 homolog D	N/A	1	0.92
PPP6C	Serine/threonine-protein phosphatase 6 catalytic subunit	N/A	1	3.28
ATG7	Ubiquitin-like modifier-activating enzyme ATG7	N/A	1	3.41
CISD2	CDGSH iron-sulfur domain-containing protein 2	N/A	1	30
OTULIN	Ubiquitin thioesterase otulin (Fragment)	N/A	1	13.64
RAB11A	Ras-related protein Rab-11A	N/A	1	7.53
COPS7A	COP9 signalosome complex subunit 7a (Fragment)	N/A	1	8
PIK3R4	Phosphoinositide 3-kinase regulatory subunit 4	N/A	1	0.66
PLEKHF2	Pleckstrin homology domain-containing family F member 2	N/A	1	3.61
FKBP4	Peptidyl-prolyl cis-trans isomerase FKBP4	N/A	1	2.18
UBE3C	Ubiquitin-protein ligase E3C	N/A	1	1.02
CMTM5	CKLF-like MARVEL transmembrane domain-containing protein 5	N/A	1	11.02
SCAMP4	Secretory carrier-associated membrane protein 4	N/A	1	4.8
AP1B1	AP-1 complex subunit beta-1 (Fragment)	N/A	1	1.38
PAK2	Serine/threonine-protein kinase PAK 2	N/A	1	1.72
UBXN1	UBX domain-containing protein 1	N/A	1	3.78
EIF3D	Eukaryotic translation initiation factor 3 subunit D	N/A	1	1.64
G6PD	Glucose-6-phosphate 1-dehydrogenase	N/A	1	1.94
CAD	Aspartate carbamoyltransferase	N/A	1	0.6
WDR26	WD repeat-containing	N/A	1	6.44

	protein 26 (Fragment)			
HSD17B11	Estradiol 17-beta-dehydrogenase 11	N/A	1	3.52
TMEM63B	CSC1-like protein 2 (Fragment)	N/A	1	1.73
YOD1	Ubiquitin thioesterase OTU1	N/A	1	2.59
ATP5F1C	ATP synthase subunit gamma, mitochondrial	N/A	1	3.02
IGBP1	Immunoglobulin-binding protein 1	N/A	1	3.24
IST1	IST1 homolog (Fragment)	N/A	1	5.42
PGM2L1	Glucose 1,6-bisphosphate synthase	N/A	1	1.77
KDELR1	ER lumen protein-retaining receptor 1	N/A	1	3.77
FLG2	Filaggrin-2	N/A	1	0.5
EXOC3	Exocyst complex component 3	N/A	1	1.95
H2BC12	Histone H2B type 1-K	N/A	1	8.73
EIF4G1	Eukaryotic translation initiation factor 4 gamma 1 (Fragment)	N/A	1	1.19
CARHSP1	Calcium-regulated heat-stable protein 1	N/A	1	7.48
GCA	Grancalcin (Fragment)	N/A	1	6.16
ENO1	2-phospho-D-glycerate hydro-lyase	N/A	1	1.84
COPS2	COP9 signalosome complex subunit 2	N/A	3	8.8
H3-3B	Histone H3	N/A	1	7.61
SEC61A2	Protein transport protein Sec61 subunit alpha isoform 2 (Fragment)	N/A	1	3.07
VIPAS39	Spermatogenesis-defective protein 39 homolog	N/A	1	1.42
RPS17	40S ribosomal protein S17	N/A	1	7.41
BPTF	Nucleosome-remodeling factor subunit BPTF (Fragment)	N/A	1	1.67
CKAP5	Cytoskeleton-associated protein 5	N/A	1	0.54
PPIAL4H	Peptidyl-prolyl cis-trans isomerase A-like 4H	N/A	1	4.27
MRPS34	28S ribosomal protein S34, mitochondrial	N/A	1	3.21
RNF126	RING-type E3 ubiquitin transferase (Fragment)	N/A	1	2.83
ETFA	Electron transfer flavoprotein subunit alpha	N/A	1	3.06
GP5	Platelet glycoprotein V	N/A	1	1.61
APMAP	Adipocyte plasma membrane-associated protein (Fragment)	N/A	1	1.71
MCM9	DNA helicase MCM9	N/A	1	0.7
SEC62	Translocation protein SEC62	N/A	1	9.41
SPATA18	Mitochondria-eating	N/A	1	1.49

	protein			
TPO	Thyroid peroxidase (Fragment)	N/A	1	8.26
AGPAT5	1-acyl-sn-glycerol-3-phosphate acyltransferase epsilon	N/A	1	14.77
CNIH4	Protein cornichon homolog 4	N/A	1	14.6
NPM1	Nucleophosmin (Fragment)	N/A	1	22.03
**Proteins presence in both groups but with an intensity ratio of at least 1.5-Fold increase**				
ATP2B4	Plasma membrane calcium-transporting ATPase 4	10.50	11.00	21.84
OSBP2	Oxysterol-binding protein	2.00	2.00	2.52

Supplementary Table 8. PfSR10 mutants primers

Name	Primers
L382A-F	5'- CAGCAAGGCGAACCTGTACGTGCTGAGC -3'
L382A-R	5'- GTACAGGTTCGCCTTGCTGATCTTATCG -3'
L387A-F	5'- GTACGTGGCGAGCATGATCATCTACTCTATC -3'
L387A -R	5'- CATGCTCGCCACGTACAGGTTCAGCTTGCTG -3'
Y392A-F	5'- GATCATCGCCTCTATCTACCTGTTTATC -3'
Y392A -R	5'- GATCATCGCCTCTATCTACCTGTTTATC -3'
C430A -F	5'- GAATATAGCCCTGTTCCTGTACTTCCTGAGC-3'
C430A -R	5'- CAGGAACAGGGCTATATTCTCGATCAGATAC-3'
L433A-F	5'- GCCTGTTCGCGTACTTCCTGAGCTACAACCTG-3'
L433A-R	5'- CAGGAAGTACGCGAACAGGCATATATTCTCG-3'
S437A-F	5'-CTTCCTGGCCTACAACCTGTACGCTAAGGTG -3'
S437A-R	5'-CAGGTTGTAGGCCAGGAAGTACAGGAACAGGC-3'
N439A-F	5'-GAGCTACGCCCTGTACGCTAAGGTGAACAAC-3'
N439A-R	5'-GTACAGGGCGTAGCTCAGGAAGTACAGGAAC-3'
Y441A-F	5'-CAACCTGGCCGCTAAGGTGAACAACGAGCTG -3'
Y441A-R	5'- CTTAGCGGCCAGGTTGTAGCTCAGGAAGTAC-3'
N445A-F	5'- CTAAGGTGGCCAACGAGCTGCTGTTTATCAG-3'
N445A-R	5'-CTCGTTGGCCACCTTAGCGTACAGGTTGTAG -3'
I451A-F	5'- CTGTTTGCCAGCGTGTGCAGCTCCATCCTG-3'
I451A-R	5'- CACGCTGGCAAACAGCAGCTCGTTGTTCAC-3'
I457A-F	5'- CAGCTCCGCCCTGAAGAACGTGTGTAGCTATC-3'
I457A-R	5'- CTTCAGGGCGGAGCTGCACACGCTGATAAAC-3'
L458A-F	5'- CTCCATCGCGAAGAACGTGTGTAGCTATC-3'
L458A-R	5'- CGTTCTTCGCGATGGAGCTGCACACGCTG-3'
D500A-F	5'- CATCTTCGCCTTCATCAAGCAATTTGTTGAC-3'
D500A-R	5'-GATGAAGGCGAAGATGATGAAAAAAAAGAAC -3'
L510A-F	5'- CATGCACGCGACTGATACACAGATCAAC-3'
L510A-R	5'-GTATCAGTCGCGTGCATGTCAACAAATTG -3'
C454A-F	5'- CAGCGTGGCCAGCTCCATCCTGAAGAACGTG-3'
C454A-R	5'-CAGGATGGAGCTGGCCACGCTGATAAACAGC -3'
F496A-F	5'-CTTTTTTGCCATCATCTTCGACTTCATCAAG -3'
F496A-R	5'-GAAGATGATGGCAAAAAAGAACAGGATCTTG-3'
K503A-F	5'-CTTCATCGCGCAATTTGTTGACATGCACCT -3'
K503A-R	5'-CAAATTGCGCGATGAAGTCGAAGATGATG -3'
T513A-F	5'-GACTGATGCACAGATCAACACCGGCTACTTCTTC -3'
T513A-R	5'-GTTGATCTGTGCATCAGTCAGGTGCATGTCAAC -3'
Y519A-F	5'-CACCGGCGCCTTCTTCTTCTGCATCATCCCTG -3'
Y519A-R	5'-GAAGAAGAAGGCGCCGGTGTTGATCTGTGTATC-3'
F520A-F	5'- CACCGGCTACGCCTTCTTCTGCATCATCCCTGTC -3'
F520A-R	5'- GAAGAAGGCGTAGCCGGTGTTGATCTGTGTATC-3'
F521A-F	5'-CTACTTCGCCTTCTGCATCATCCCTGTCACAATCATC -3'
F521A-R	5'- GCAGAAGGCGAAGTAGCCGGTGTTGATCTGTGTATC-3'
F522A-F	5'- CTTCTTCGCCTGCATCATCCCTGTCACAATC-3'
F522A-R	5'- GATGCAGGCGAAGAAGTAGCCGGTGTTGATC-3'
C523A-F	5'- CTTCTTCGCCATCATCCCTGTCACAATCATC-3'
C523A-R	5'- GATGATGGCGAAGAAGAAGTAGCCGGTGTTG-3'
I525A-F	5'-CTGCATCGCCCCTGTCACAATCATCTACAGC -3'
I525A-R	5'- GACAGGGGCGATGCAGAAGAAGAAGTAGCCG-3'
T528A-F	5'-CATCCCTGTCGCAATCATCTACAGCATCATCTATATC-3'
T528A-R	5'-GCTGTAGATGATTGCGACAGGGATGATGCAGAAG-3'
I529A-F	5'- CATCCCTGTCACAGCAATCTACAGCATCATCTATATC-3'
I529A-R	5'- GCTGTAGATTGCTGTGACAGGGATGATGCAGAAG-3'
I534A-F	5'-CATCTACAGCATCGCATATATCTGGGTGTTCAC-3'
I534A-R	5'-CAGATATATGCGATGCTGTAGATGATTGTGACAG-3'
W537A-F	5'-CATCATCTATATCGCAGTGTTCACATCTGCCAGC-3'
W537A-R	5'-GATGTGAACACTGCGATATAGATGATGCTGTAGATG-3'
F579A-F	5'-GATTGCCGCCATCATCGACATCGTCGTGATG-3'

F579A-R	5'-GTCGATGATGGCGGCAATCACGCTGAAGAGC-3'
M586A-F	5'-CGTCGTGGCGCTGTACGTGGATAACAGCATC-3'
M586A-R	5'-GTACAGCGCCACGACGATGTCGATGATGAAG-3'
I587A-F	5'-GTGATGGCGTACGTGGATAACAGCATCTGGAAC-3'
I587A-R	5'-CACGTACGCCATCACGACGATGTCGATGATGAAG-3'
N591A-F	5'-GTGGATGCCAGCATCTGGAACCTGAAGAATTACCTG-3'
N591A-R	5'-GATGCTGGCATCCACGTACAGCATCACGACGATGTC-3'
Y599A-F	5'-GAAGAATGCCCTGTCTGAAGGCATCATCTCTTGTCTG-3'
Y599A-R	5'-CAGACAGGGCATTCTTCAGGTTCCAGATGCTGTTCCAG-3'
E602A-F	5'-CTGTCTGCAGGCATCATCTCTTGTCTGTTCCTG-3'
E602A-R	5'-GATGCCTGCAGACAGGTAATTCTTCAGGTTCCAG-3'
S606A-F	5'-CATCATCGCTTGTCTGTTCCTGATCATCCTGACCGCCATG-3'
S606A-R	5'-CAGACAAGCGATGATGCCTTCAGACAGGTAATTCTTCAG-3'
F609A-F	5'-CTTGTCTGGCCCTGATCATCCTGACCGCCATG-3'
F609A-R	5'-GATCAGGGCCAGACAAGAGATGATGCCTTCAG-3'
L610A-F	5'-GTCTGTTCGCGATCATCCTGACCGCCATGTTC-3'
L610A-R	5'-CAGGATGATCGCGAACAGACAAGAGATGATGC-3'
F574A-F	5'-CACACTGCTCGCAAGCGTGATTGCCTTCATC-3'
F574A-R	5'-CAATCACGCTTGCGAGCAGTGTGAAAATCAG-3'
D582A-F	5'-GCCTTCATCATCGCAATCGTCGTGATGCTGTACGTGGATAAC-3'
D582A-R	5'-CATCACGACGATTGCGATGATGAAGGCAATCACGC-3'
Y588A-F	5'-GATGCTGGCCGTGGATAACAGCATCTGGAACCTG-3'
Y588A-R	5'-GTTATCCACGGCCAGCATCACGACGATGTCGATG-3'
N595A-F	5'-CATCTGGGCCCTGAAGAATTACCTGTCTGAAGGC-3'
N595A-R	5'-CTTCAGGGCCCAGATGCTGTTATCCACGTACAGC-3'

Supplementary Table 9. PfSR10 FlAsH mutants primers

Name	Primers
PfSR10-Nluc-F1	5’-CGGGAAGCGCTGGTAGTGCGCTGAACCTGTACGTGCTGAGCATGATC-3’
PfSR10-Nluc-R1	5’-CGAAATCTTCGAGTGTGAAGACCATGGCCTTATCGTCGTCATCCTTGTAG -3’
PfSR10-Nluc-F2	5’-GTCTTCACACTCGAAGATTTCGTTGGGGACTGGCGACAGACAGCCG-3’
PfSR10-Nluc-R2	5’-CGCACTACCAGCGCTTCCCGCCAGAATGCGTTCGCACAG-3’
438F	5’-TGCTGTCCAGGTTGTTGCAACCTGTACGCTAAGGTGAACAACGAGC-3’
438R	5’-GTTGCAACAACCTGGACAGCAGTAGCTCAGGAAGTACAGGAACAGGC-3’
441F	5’-GCTGTCCAGGTTGTTGCGCTAAGGTGAACAACGAGCTGCTGTTTATC-3’
441R	5’-GCGCAACAACCTGGACAGCAGTACAGGTTGTAGCTCAGGAAGTAC-3’
506F	5’-GCTGTCCAGGTTGTTGCGACATGCACCTGACTGATACACAGATCAAC-3’
506R	5’-GTCGCAACAACCTGGACAGCAAACAAATTGCTTGATGAAGTCGAAGATG-3’
514F	5’-GCTGTCCAGGTTGTTGCATCAACACCGGCTACTTCTTCTTCTGCATC-3’
514R	5’-GATGCAACAACCTGGACAGCACTGTGTATCAGTCAGGTGCATGTCAAC-3’
592F	5’-GCTGTCCAGGTTGTTGCATCTGGAACCTGAAGAATTACCTGTCTG-3’
592R	5’-GATGCAACAACCTGGACAGCAGCTGTTATCCACGTACAGCATCACG-3’
596F	5’-GCTGTCCAGGTTGTTGCAAGAATTACCTGTCTGAAGGCATCATCTC-3’
596R	5’-CTTGCAACAACCTGGACAGCACAGGTTCCAGATGCTGTTATCCACG-3’
